# A novel bioinformatic approach reveals cooperation between Cancer/Testis genes in basal-like breast tumors

**DOI:** 10.1038/s41388-024-03002-7

**Published:** 2024-03-11

**Authors:** Marthe Laisné, Brianna Rodgers, Sarah Benlamara, Julien Wicinski, André Nicolas, Lounes Djerroudi, Nikhil Gupta, Laure Ferry, Olivier Kirsh, Diana Daher, Claude Philippe, Yuki Okada, Emmanuelle Charafe-Jauffret, Gael Cristofari, Didier Meseure, Anne Vincent-Salomon, Christophe Ginestier, Pierre-Antoine Defossez

**Affiliations:** 1 Université Paris Cité, CNRS, Epigenetics and Cell Fate, F-75013 Paris, France; 2 CRCM, Inserm, CNRS, Institut Paoli-Calmettes, Aix-Marseille University, Epithelial Stem Cells and Cancer Laboratory, Equipe Labellisée LIGUE Contre le Cancer, Marseille, France; 3 Platform of Experimental Pathology, Department of Diagnostic and Theranostic Medicine, Institut Curie-Hospital, 75005 Paris, France; 4 Department of Pathology, Institut Curie, 26 Rue d’Ulm, 75005 Paris, France; 5 Université Côte d’Azur, Inserm, CNRS, IRCAN, Nice, France; 6 Institute for Quantitative Biosciences, The University of Tokyo, Tokyo, Japan

**Keywords:** Oncogenes, Cancer genomics

## Abstract

Breast cancer is the most prevalent type of cancer in women worldwide. Within breast tumors, the basal-like subtype has the worst prognosis, prompting the need for new tools to understand, detect, and treat these tumors. Certain germline-restricted genes show aberrant expression in tumors and are known as Cancer/Testis genes; their misexpression has diagnostic and therapeutic applications. Here we designed a new bioinformatic approach to examine Cancer/Testis gene misexpression in breast tumors. We identify several new markers in Luminal and HER-2 positive tumors, some of which predict response to chemotherapy. We then use machine learning to identify the two Cancer/Testis genes most associated with basal-like breast tumors: HORMAD1 and CT83. We show that these genes are expressed by tumor cells and not by the microenvironment, and that they are not expressed by normal breast progenitors; in other words, their activation occurs de novo. We find these genes are epigenetically repressed by DNA methylation, and that their activation upon DNA demethylation is irreversible, providing a memory of past epigenetic disturbances. Simultaneous expression of both genes in breast cells in vitro has a synergistic effect that increases stemness and activates a transcriptional profile also observed in double-positive tumors. Therefore, we reveal a functional cooperation between Cancer/Testis genes in basal breast tumors; these findings have consequences for the understanding, diagnosis, and therapy of the breast tumors with the worst outcomes.

## Introduction

Cancer cells undergo massive genetic and epigenetic changes relative to their normal progenitors [1, 2]. The advances in genomics and epigenomics have yielded an ever more complete picture of these abnormalities, and drawn accurate molecular portraits of different tumor types (for example: [3–5]). The large number of samples examined in public cohorts increase statistical power, yet parsing out the driver from passenger events remains far from trivial [6].

Besides mutations in the coding sequence, gene function can also be altered by changes in the level of gene expression, as a consequence of genetic and epigenetic modifications in tumors. Genes can be turned off by deletions, alterations in their control elements such as enhancers, or changes in the transcriptional machinery. Conversely, they can become overexpressed by amplification, gain of enhancers, or expression of transcriptional activators, among other possibilities. Genes that are frequently turned on in a tumor type are useful as biomarkers [7, 8]; in some instances, their expression can inform prognosis and choice of treatment. Some of these overexpressed genes also play a direct functional role in the tumor cells, and therefore represent therapeutic targets. HER2 is a prime example: the gene’s overexpression marks a specific subtype of breast tumors, and highly efficient therapeutic antibodies have been generated against this target [9, 10].

HER2 is expressed by normal breast cells, so its overexpression in breast tumors is just the amplification of a pre-existing expression pattern. However, tumor cells can also deviate radically from their ancestral gene expression pattern and turn on genes that are normally activated in other tissue types or at other developmental stages [11]. For instance, various tumor types in men and women express genes that are typical of the placenta [12, 13]. Within this broad framework of ectopic gene reactivation in tumors, one class of genes bears particular conceptual interest and therapeutic promise: Cancer/Testis (C/T) genes.

As their name implies, Cancer/Testis gene expression is usually restricted to the male germline, but it can be reactivated in tumors (in both male and female patients). As their expression is not present in any normal somatic cells, they are remarkable biomarkers for tumors [14]. Additionally, as the testis is in immune sanctuary in men [15, 16], and because testicular genes are not typically expressed in women, their expression in tumors opens an excellent possibility for immunotherapy [17, 18]. Finally, Cancer/Testis genes may be oncogenes in their own right and are potential drug targets for therapy [19].

Breast cancer is the most common cancer in women, both in developed and developing countries, and breast malignancies killed almost 700,000 women worldwide in 2020 [20, 21]. It has long been understood that breast tumors form a heterogeneous ensemble, with at least five distinguishable subtypes: normal-like, Luminal A, Luminal B, HER2-positive, and basal-like [7, 22]. Within those groups, basal-like tumors could themselves contain distinct subtypes and are associated with the worst prognosis, having few dedicated therapies [23].

Cancer/Testis genes have been investigated as potential biomarkers, oncogenes, and targets in breast cancer, with promising results [24–28]. To build on these investigations, we undertook an unbiased analysis of publicly available expression data with a new bioinformatic approach. This led us to discover several new markers associated with different breast tumor subtypes. Our cohort of in situ tumors establishes that Cancer/Testis gene activation is an early event in tumorigenesis, and that there is no switch of their expression pattern between early and more established tumors. We then focus on the two genes whose expression is most highly associated with basal breast tumors: HORMAD1 and CT83. We show that these genes are not expressed by healthy cell progenitors but activate de novo in the tumor cells. We demonstrate that loss of methylation is sufficient to reactivate both genes, and that an initial activation event is sufficient to trigger persistent expression. Most basal tumors express at least one of the two genes, but those that express both have a significantly worse outcome, hinting at a cooperative effect. Using breast cells in culture, we prove that the two genes synergize to modulate stemness and initiate a transcriptional signature that is also found in basal tumors expressing HORMAD1 and CT83 simultaneously. These findings advance our conceptual understanding of Cancer/Testis genes in breast cancer and have practical implications for diagnosis and treatment.

## Results

### A custom bioinformatic approach identifies the Cancer/Testis genes most associated with breast tumors

The first step of our study was to establish an exhaustive list of C/T genes, containing those described in three independent publications, for a total of 1350 genes [12, 29, 30]. Our second resource was genomics data, including RNA-seq, from The Cancer Genome Atlas (TCGA), covering 1090 tumor samples and 113 healthy juxta-tumoral mammary samples.

**Fig. 1:**
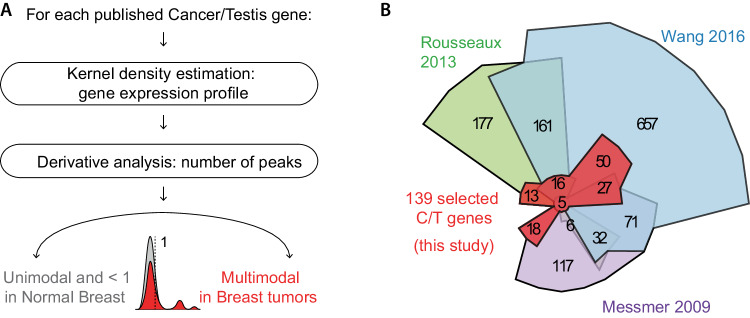
A custom bioinformatic approach identifies the Cancer/Testis genes most associated with breast tumors. **A** Schematic description of the bioinformatic pipeline. We depict the expression profile of a gene that passed the screen: it has a unimodal, zero-centered profile in normal tissue, and a multimodal profile in breast tumors. **B** Chow-Ruskey diagram showing the intersection between previously published C/T gene lists and the C/T genes that were selected for our study.

We then established a custom bioinformatic approach to identify C/T genes that show reactivation in breast tumors. An ideal biomarker should have little or no expression in healthy samples but high expression in at least some of the tumors: these properties are reflected mathematically in a zero-centered, single-mode density function in healthy breast samples, and a multi-mode density function with one or more non-zero maxima in tumor samples, reflecting one or more groups of tumors that have activated this gene. Such profiles can be detected automatically by examining changes in the derivative of the density function (Fig. 1A).

Implementing this idea, we created a two-step pipeline in which we first determined the distribution of expression for each C/T gene in both healthy mammary samples and breast tumors, then smoothed these distributions using kernel density estimation. As it is crucial to not overfit or oversmooth expression values, we systematically tested multiple values for the bandwidth parameter using positive and negative controls, (data not shown) and then selected a balanced value (bandwidth = 0.7). By analyzing the derivative of the distribution function, we obtained the number of distinct peaks, allowing us to focus on the C/T genes not expressed in healthy mammary samples (unimodal expression profile centered on 0 according to kernel density estimation), but activated in some breast tumor samples (multimodal expression profile).

Our method complements previously used approaches (for example: [30, 31]) in that it is orthogonal, less calculation-intensive, flexible, sensitive, and unaffected by the dynamic range of the data. Of note, this unbiased scheme is not restricted to C/T genes and could be broadly used to identify other genes that show abnormal expression in tumor samples compared to matched normal juxta-tumor tissues, such as potential tumor suppressor genes or oncogenes (Fig. S1A–C). With this approach, we defined a highly selective list of 139 C/T genes with abnormal expression profiles in breast tumors compared to the normal breast (Fig. 1B, Supplementary Table 1). The examination of GTEx RNA-seq data confirmed that these 139 genes are expressed in the human germline, but not in the breast (or other healthy tissues, Fig. S1D). Therefore, the reactivation seen in tumors is a pathological event.

### Cancer/Testis gene expression accurately discriminates breast cancer subtypes; identification of the 6 most informative genes

**Fig. 2:**
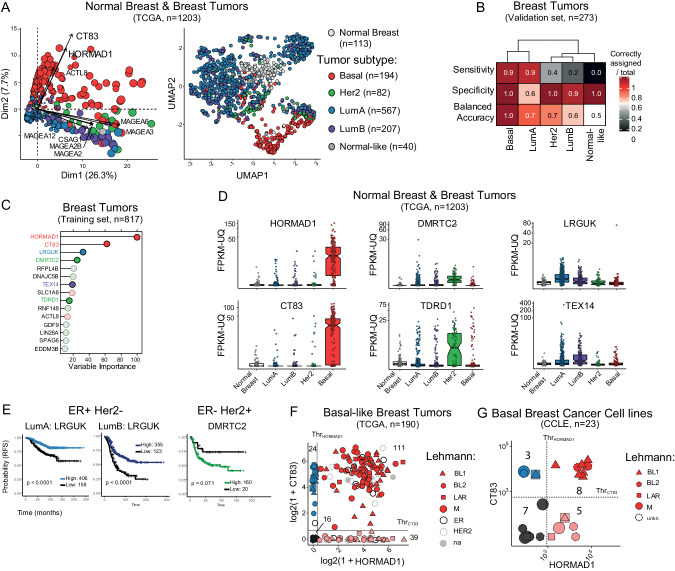
Cancer/Testis gene expression accurately discriminates breast cancer subtypes; identification of the 6 most informative genes. **A** Multidimensional analysis of TCGA breast tumor and healthy samples based on expression of the 139 selected C/T genes. Each dot represents a sample; the color code corresponds to breast cancer subtype. Left: Principal Component Analysis, dot sizes are proportional to the quality of representation in PC1/PC2 space. The C/T genes best correlated to PC1/PC2 are represented. Right: Uniform Manifold Approximation and Projection (UMAP). **B** Confusion matrix for breast tumor samples in the validation cohort (25% of the samples, randomly selected from the TCGA breast tumors), using the best Random Forest model. This model was established after a 500-tree training on the discovery cohort (75%), based on the expression level of the 139 C/T genes. **C** Top 15 most important variables in the best Random Forest model for breast cancer subtype prediction. The color of the gene name indicates the tumor type most associated. **D** Expression levels for 6 subtype-specific C/T genes in the breast TCGA cohort according to breast cancer subtype. **E** Relapse-free survival curves for ER+ Her2- or ER- Her2+ breast cancer patients according to LRGUK and DMRTC2 expression. The left two panels show survival curves for Luminal A and Luminal B tumors. The right panel shows the survival curve according to Her2-specific C/T gene DMRTC2. **F** Co-expression of HORMAD1 and CT83 based on RNA-seq analysis (log2 FPKM-UQ) in basal-like breast tumor samples from the TCGA. Thresholds for positive or negative expression are calculated based on the corresponding gene expression profile in tumors at the second inflexion point of the representative curve. The number of tumors belonging to each category are shown. Basal-like subculturing according to Lehmann’s classification is depicted. **G** Co-expression of HORMAD1 and CT83 based on RNA-seq analysis (FPKM-UQ) in basal-like breast cancer cell lines from the CCLE database. Same analysis as in F.

To determine whether the expression of certain members of our 139-gene list was associated with specific subtypes of breast tumors, we applied Principal Component Analysis (PCA) on TCGA data using the subtype annotations provided for each tumor (Fig. 2A). A visual inspection suggested that tumor types could indeed be separated based on C/T gene expression (Fig. 2A), with a distinct group of basal-like tumors, for instance. These distinct clusters formed again when the tumors were classified based on their anatomohistological subtype rather than their transcriptome-defined subtype (Fig. S2A) and they remained visible when integrating more informative Principal Components through UMAP analysis (Figs. 2A and S2A). We thus hypothesized that the pattern of expression of the C/T genes in our list might suffice to stratify breast tumors by subtypes.

To test this hypothesis, we used a machine learning approach, establishing a random forest model on a training set of TCGA breast tumors (75% of all samples, *n* = 817) and testing the best model on the remaining tumors (*n* = 273). This model could very effectively identify basal tumors with high sensitivity (0.9) and high specificity (1.0), leading to a balanced accuracy nearing 100% (Fig. 2B). Again, similar results were found when the tumors were classified anatomopathologically, rather than transcriptionally (Fig. S2B). The specificity scores for Luminal B and Her2 subtypes were high (1.0 and 0.9, respectively), but the sensitivity was lower (0.4 and 0.2) (Fig. 2B). This discrepancy could be explained by some tumors in these groups not expressing any C/T genes, leading to a lack of available information for the prediction.

Using the best random forest model, we ranked the 139 C/T genes according to their predictive value; the top 15 C/T genes are depicted in Fig. 2C (and Fig. S2C for the analysis carried out with anatomopathological stratification). The two best predictors, HORMAD1 and CT83, are strongly associated with basal breast tumors: of the 190 basal-like breast tumors, 89% expressed either HORMAD1 or CT83, compared to only 13% of Her2-amplified, 6% of Luminal B, and 2% of Luminal A tumors (Fig. 2D). Using the histological classification of breast tumors, we found the same result: HORMAD1 and CT83 are the two best predictors of triple-negative breast cancers within C/T genes (Fig. S2B–D). These results are consistent with several previous reports that have associated HORMAD1 or CT83 expression with basal tumors [32–36], validating our approach. Analysis of an independent dataset [37] gave additional support to our findings, demonstrating that HORMAD1 and CT83 are the best predictors of triple-negative breast tumor subtype (Fig. S2 E, F). HORMAD1, a gene on human chromosome 1q21.3, is physiologically expressed by preleptotene spermatocytes [38] and regulates meiotic progression. CT83, on the other hand, is located on human chromosome region Xq23 and is expressed in mature sperm according to scRNA-seq data analysis [39], yet its precise reproductive function remains unknown.

The expression of two other markers, DMRTC2 and TDRD1, is associated with Her2-positive tumors (Fig. 2C, D), but the association is looser than that of HORMAD1/CT83 with basal tumors. Throughout spermatogenesis, DMRTC2 has essential functions during pachytene [40], whereas TDRD1 interacts with piRNAs and Piwi proteins to promote silencing [41]. To the best of our knowledge, neither DMRTC2 nor TDRD1 have been previously linked to breast tumors in general, nor to the HER-2 positive subtype in particular.

Lastly, we found two markers, LRGUK and TEX14, for which expression tends to mark luminal tumors (Fig. 2D). LRGUK is involved in diverse aspects of sperm assembly, including the microtubule-based shaping of spermatozoa [42]; it was more frequently overexpressed in luminal A breast tumors (Fig. 2D). As for TEX14, a factor necessary for intracellular bridges in germ cells [43], it marked luminal B breast cancers, as well as luminal A tumors to a smaller extent (Fig. 2D). While TEX14 was previously linked to basal breast tumors [44], we believe our study presents the first report demonstrating its more prevalent expression in Luminal tumors, especially of the more aggressive B subtype, and we are unaware of any publications linking LRGUK to breast tumors in general, nor to Luminal tumors in particular.

We next tested whether the associations we detected using tumor expression data also held true with cancer cell lines. As tumors are heterogeneous and consist of a mixture of cell types, including tumor cells and cells from the microenvironment, we asked if the expression of C/T genes detected in bulk RNAseq are due to their activation in tumor cells themselves. Detecting high expression of C/T genes in tumor-derived cell lines would be a strong cue for expression of the C/T genes in the tumor cells themselves. For this, we determined the expression level of the six markers described above in all the breast cell lines found in the Cancer Cell Line Encyclopedia (CCLE, Fig. S2G). We observed a good general agreement between tumors and cell lines of the same subtype. For instance, HORMAD1 and/or CT83 were highly expressed in the basal cell lines such as MDA-MB-436, MDA-MB-468, and HCC1599, but not in Luminal or Her2-positive cells. DMRTC2 and/or TDRD1 expression marked HER2-positive lines like AU565 or SKBR3. Finally, a typical Luminal A line, MCF7, expressed LRGUK and had the highest TEX14 levels. These results validate our findings, and also suggest that the overexpression of C/T genes detected in bulk RNAseq is due at least in part to the abnormal activation of these genes in tumor cells.

Finally, we asked whether the expression of these C/T genes could distinguish, within a breast cancer subtype, tumors with a different prognosis or therapeutic response. We examined relapse-free survival at more than 10 years, on a large panel of breast tumors of known subtype [45]. The activation of LRGUK in Luminal A or Luminal B tumors was an indicator of a good prognosis (Fig. 2E). Furthermore, this activation correlated with a better response to anthracyclines (used in standard-of-care chemotherapy regimens), although the trend failed to reach significance (Fig. S2H). Still in the luminal subtype, no significant association was found between survival and TEX14 expression (Fig. S2I), raising the possibility that the activation of certain C/T genes may be a neutral event, with no association with a particular phenotype, and not conferring a specific advantage or disadvantage, at least at this stage of tumor development.

For Her2-positive tumors, the expression of TDRD1 was not statistically linked to survival (Fig. S2J). In the same tumors, DMRTC2 expression tended to associate with poorer survival, however the trend did not reach statistical significance, maybe because the size of the DMRTC2-negative group was small (*n* = 20), in line with the prevalent re-expression of DMRTC2) in Her2-positive tumors (Fig. 2E). To detect other potentially useful characteristics of these tumors, we examined their immunological signature with the Immunoscore tool [46] (Fig. S2K): those with high DMRTC2 were more “hot”, i.e. more infiltrated, but also more immunosuppressive (high FOXP3 activation). Therefore, they might be attractive candidates for treatment with immune checkpoint inhibitors [47]. As far as we are aware, these associations are new and may be helpful for prognosis and treatment choice.

In the TCGA cohort, ~90% of basal-like tumors expressed HORMAD1 or CT83 at the RNA level, and ~60% expressed both (Fig. 2F). Basal-like tumors are a heterogeneous ensemble, but tumors expressing both HORMAD1 and CT83 tended to form a more homogeneous set, with fewer distinct anatomopathological groups and a reduced number of molecular signatures (Fig. S2N and Supplementary Table 2). Using the Lehmann classification [48], we found double-positive tumors in all subgroups (Fig. 2F). In breast cancer cell lines as well, 70% of basal-like cell lines from CCLE were positive for HORMAD1 or CT83 and 35% for both (Fig. 2G).

### The activation of subtype-specific cancer/testis genes occurs in tumoral cells early during tumorigenesis and persists in metastasis

**Fig. 3:**
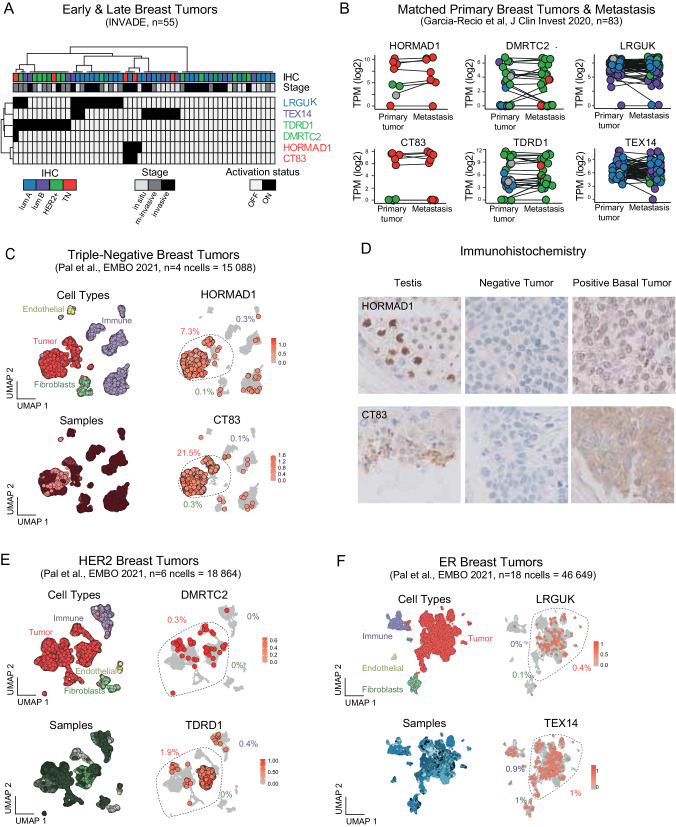
The activation of subtype-specific Cancer/Testis genes occurs in tumoral cells early during tumorigenesis and persists in metastasis. **A** Expression of the indicated C/T genes in early (in situ or microinvasive) and late (invasive) tumors of the INVADE cohort. Hierarchical clustering of early and late breast tumor samples based on expression of the top 6 C/T genes previously described. A C/T gene is depicted as activated (black box) if its expression value is above the background expression threshold. Tumor subtypes are differentiated by color. **B** Expression level of breast-cancer specific C/T genes in matched primary tumor and metastasis. Color code reflects tumor subtype, as in (**A**). **C** UMAP representation of a scRNA-seq study on four triple-negative breast tumors (GSE161529). Each dot is either a tumor cell or a cell from the tumor microenvironment. Epithelial clusters of tumor cells are highlighted, HORMAD1 and CT83 expressions are shown. **D** Immunohistochemistry of HORMAD1 (top) or CT83 (bottom) in normal testis and in two breast cancer samples showing no (left) or positive (right) expression. **E** UMAP representation of a scRNA-seq study on 4 HER2 breast tumors (GSE161529). Each dot is either a tumor cell or a cell from the tumor microenvironment. Epithelial clusters of tumor cells are highlighted, DMRTC2 and TDRD1 expression is shown. **F** UMAP representation of a scRNA-seq study on 18 ER breast tumors (GSE161529). Each dot is either a tumor cell or a cell from the tumor microenvironment. Epithelial clusters of tumor cells are highlighted, TEX14 and LRGUK expression is shown.

The association we report between expression of specific C/T genes and breast cancer subtypes was found in an unbiased analysis of the TCGA breast tumor set, but this set primarily contains mid- and late-stage malignancies. A practically and conceptually important question is whether these markers are already expressed at the early stages of tumorigenesis. To further explore this, we used RNA-seq analysis of early tumors (in situ and microinvasive) and invasive breast carcinomas of different subtypes (*n* = 55, our INVADE cohort, Fig. 3A). Twenty-four of the 35 early tumors (68%) expressed at least one of the markers, while 11 out of 20 invasive tumors (55%) did so. The association between marker and tumor type was generally respected: for instance, LRGUK was expressed in 14 tumors, of which 11 were luminal (*p*-value = 2 · 10^−7^), seven of those being early-stage, and the remaining four were invasive. TDRD1 was expressed in 12 samples, of which seven were HER2-positive (*p*-value = 2 · 10^−4^), and six out of those seven were early-stage. DMRTC2 was not found in any early HER2-positive samples, possibly indicating that its expression is induced later in tumorigenesis. The expression of HORMAD1 and CT83 was rare, which is unsurprising as basal-like tumors are rarely diagnosed at early stages.

We next asked whether the expression of these six markers is also present in metastases from breast cancer, depending on the subtype of the primary tumor. For this purpose, we used RNA-seq data from 83 primary tumors matched with the corresponding metastases (Fig. 3B). By separating the tumors according to PAM50 subtype, we observed two things: 1. All six markers are expressed by the corresponding subtype, including when the cancer is metastatic; 2. In the majority of cases, if the primary tumor expresses one of the six C/T markers, the metastases from this tumor will also express it. For HORMAD1 and CT83, we further validated this result on an independent dataset (Fig. S3A). Moreover, in the case of multiple metastatic seeding sites, metastases also maintained their expression of HORMAD1 or CT83 independently of the seeding site (Fig. S3B), implying that the expression of these C/T genes is maintained during metastatic progression. We hypothesized that the clones within the primary tumor that will evolve to form metastases are those which already express these C/T genes, further enhancing the potential of these genes as biomarkers.

Using single-cell RNA-seq data, we zoomed into intra-tumoral heterogeneity to understand whether C/T genes are indeed expressed by cancer cells (and not by cells from the microenvironment) and to analyze the clonality of this expression. We utilized a complete study on 39 breast tumors of different subtypes [49], selecting high quality cells and analyzing each tumor subtype separately. After dimensional reduction, cells formed clusters according to cell types (Fig. S3C). Of the four analyzed triple-negative breast tumors (Fig. 3C) we found several HORMAD1 (in 3/4 tumors) and CT83 (in 4/4 tumors) positive cells: they fall primarily into the tumor cell cluster. Interestingly, one of the four tumors shows two subclusters of tumor cells: one subcluster is positive for HORMAD1 yet the second is not, revealing that intra-tumoral heterogeneity may exist in some samples. Increasing the resolution of our analysis, we then used full-length scRNA-seq of triple-negative tumors [50] and again identified robust expression of HORMAD1 and CT83 in tumor cells (Fig. S3D). Within any given tumor approximately 20–40% of individual cancer cells express either HORMAD1 or CT83, and around 5–20% express both.

We then sought to confirm and complement these transcriptional analyses with immunohistochemistry (IHC). We screened antibodies and experimental conditions until we arrived at combinations under which the IHC pattern observed on human testis sections matched the results of single-cell RNA-seq in the same organ [39]. With these conditions, we could observe nuclear staining for HORMAD1 specifically in preleptotene spermatocytes, and staining in mature spermatozoids for CT83 (Fig. 3D). Using the same conditions on 99 tumor sections of mixed types, we verified that most triple-negative tumors (34 out of 40, 85%) expressed HORMAD1 and/or CT83 (Fig. S3E), and this activation is specific to the triple-negative subtype (*p*-value < 10^−4^). In the positive tumors, staining for HORMAD1 was predominantly nuclear, present in most or all tumor cells, and seemed absent from non-tumor cells of the microenvironment. CT83 staining was cytoplasmic but similarly marked most tumor cells, and few or no cells of the microenvironment (Fig. 3D).

For the HER2-related C/T markers DMRTC2 and TDRD1, only one tumor in the analyzed dataset was positive for TDRD1 (Fig. 3E). The expression in this tumor is due to the activation of TDRD1 primarily in tumor cells, with no obvious subclonality. The results for DMRTC2 were inconclusive. Finally, the expression pattern for the ER-related markers was more precarious, showing only a minority of tumor cells expressing either TEX14 or LRGUK (Fig. 3F), with a significant contribution of cells from the microenvironment for TEX14 expression.

The results from these datasets prompt several important conclusions: (1) the activation of C/T genes can be an early event during tumorigenesis, detectable within in situ tumors, (2) the type of C/T genes activated in a tumor is consistent between early and later-stage tumors, indicating there is no switch in expression, and (3) HORMAD1 and CT83 prove to be the most promising markers due to their association with the most deadly subtype of breast cancer and their robust pattern of expression (RNA and protein) in tumor cells detected at the single-cell level.

### Single-cell RNAseq reveals the expression of the C/T markers in rare cells of the normal breast

As our prior results showed that all six C/T genes of interest are present in early tumors, we next investigated whether their activation is tumorigenesis-dependent, or if it occurs in rare cells within healthy tissue. If the latter is true, this activation could be a marker of plasticity of these few cells more likely to be transformed. Another hypothesis, compatible with the first scenario, would be that the early expression of C/T genes and the activity of the resulting proteins could facilitate tumorigenesis.

**Fig. 4:**
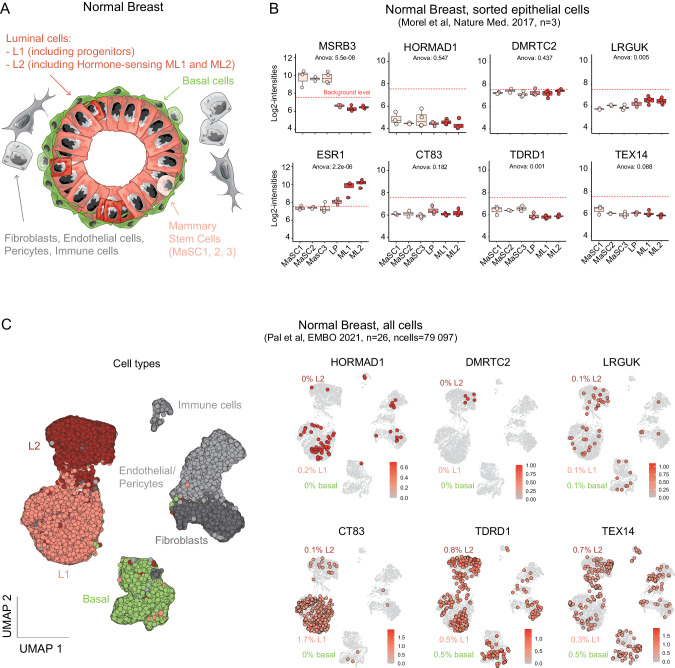
Single-cell RNA-seq reveals the expression of C/T genes in rare cells of the normal breast. **A** Schematic representation of the mammary gland, with the different epithelial and non-epithelial cell types indicated. **B** HORMAD1 and CT83 expression in sorted healthy mammary cells. The red dotted line represents the threshold for gene expression detection. MaSC mammary stem cell, LP luminal progenitor, ML mature luminal cell. **C** Left: scRNAseq of healthy breast samples from 26 healthy mammary glands (GSE161529) showing cell subtypes. Right: normalized expression of breast-cancer-specific C/T genes. Positive cells are emphasized, the percentage of positive cells in each cluster is indicated.

To delineate the origin of the C/T genes’ activation, we dove further into the different epithelial subtypes that compose the mammary gland (Fig. 4A). Here we utilized RNA expression data obtained on healthy cells sorted from reduction mammoplasties, where markers were used to FACS-sort stem cells, luminal progenitors, and mature luminal cells (Fig. 4B, [51]). Within this data, known genes displayed the expected expression pattern [51, 52]; for example, MSRB3 was expressed in stem cells but not more differentiated cells, whereas ESR1 had the opposite pattern (Fig. 4B). In contrast, none of the six C/T markers were detectably expressed in any of the sorted cell populations (Fig. 4B). In particular, HORMAD1 and CT83 were not detectably expressed in luminal progenitors, which are the proposed cells of origin for basal tumors [53, 54]. Therefore, from this bulk analysis, expression of the six C/T genes of interest in breast tumors does not seem to merely reflect pre-existing expression in any of the canonical cell subtypes from the mammary gland.

We investigated this question further using single-cell RNA-seq data from normal human breast samples, after FACS-enrichment for epithelial cells. Using a combination of dimensional reduction, unsupervised clustering approaches, and previously known markers, we were able to separate the three distinct epithelial cell types, one basal and two luminal cell types called secretory (Luminal 1 (L1), containing luminal progenitors) and hormone-responsive (Luminal 2, or L2) (Fig. S4A). The expression of Luminal (Krt18, LTF, AGR2) or Basal (Krt14) genes marked the expected populations (Fig. S4A), with no contamination from immune and stromal cells (Fig. S4A). Surprisingly, we detected some normal epithelial cells expressing C/T markers (Fig. S4B, red dots), however these cells were very rare: for example, only 12 out of 23,007 total cells expressed HORMAD1 and/or CT83, which is consistent with the lack of detection in the sorted cell populations of Fig. 4A. Interestingly, some of the C/T genes are expressed by specific cell subtypes (e.g. HORMAD1 and CT83 by L1 luminal cells), while others seem to be activated by several cell types (e.g. LRGUK, TEX14).

As this result was unexpected, it was necessary to strengthen it using an independent dataset, preferably using as many cells as possible. Utilizing a study containing nearly 80,000 cells derived from 15 mammoplasties [49], we were able to again identify the main cell types from the mammary gland (Figs. 4C and S4C, D). With this dataset containing four times as many cells as the previous one, we identified many more cells positive for the six C/T markers. Of these markers, HORMAD1 and CT83 differ in that they are activated primarily by L1 luminal cells, as before (Fig. 4C), however we did not detect any cell showing co-expression of these genes. This finding could be explained by the low number of events detected, combined with the dropout probability inherent to scRNAseq, as well as a possible counter-selection of this event in the healthy mammary gland. DMRTC2, on the other hand, is activated only by very few cells, all belonging to the L2 compartment (Fig. 4C). Here we find an association with the “reservoir” subtypes of basal-like and HER2 tumor-origin cells. In contrast, TDRD1, LRGUK, and TEX14 can be activated in virtually any cell of the mammary gland, including non-epithelial cells (Fig. 4C). For TEX14, this result is consistent with the heterogeneous expression patterns observed in tumors, where the tumor microenvironment also expresses this C/T gene (Fig. 3F).

We then tried to identify differentially expressed genes in C/T-expressing cells, compared to negative cells belonging to the same cell type, but we failed to identify any robust changes. This negative result may indicate that C/T gene activation is a neutral event for healthy cells and reflects only some alteration of transcription regulation, but it could also be an artifact due to the low number of positive cells identified.

These bioinformatic analyses provide clear evidence that, of the various C/T genes identified, HORMAD1 and CT83 are the most compelling as potential biomarkers for basal-like breast tumors due to their basal tumor-specific expression. Another surprising finding is their early activation in a small subset of specific cells in the healthy mammary gland, which is not shown for the other four C/T genes. This early activation event prompts the question of whether this expression predisposes cells to become transformed or is a marker of transcriptional and possibly epigenetic abnormalities of more plastic cells. We focused on these two genes to decipher whether their activation is linked to epigenetic alteration, and if their expression can induce functional changes in mammary epithelial cells.

### Activation of HORMAD1 and CT83 in tumors involves epigenetic alterations

To begin uncovering the role of HORMAD1 and CT83 expression in basal-like breast tumors, we first wanted to understand how this aberrant expression becomes induced. Basal-like tumors are genetically unstable [55], so we examined whether HORMAD1 and CT83 overexpression could be due to gene amplification. We found two results arguing against this possibility. First, there were no correlations between Copy Number Variation (CNV) and mRNA levels for HORMAD1 or CT83 in basal tumors (Fig. S5A). Second, if the genes’ overexpression were due to an amplification of locus, then we would expect to see a positive correlation between the expression of HORMAD1 and its two adjoining genes (GOLPH3L, 1 kb away, and CTSS, 9 kb away), and/or between CT83 and its contiguous gene SLC6A14 (250 base pairs away). We failed to detect any such correlation, whereas the expression of a gene known to undergo amplification and used as a positive control in the analysis, ERBB2, correlated positively with the expression of the neighboring gene PGAP3 (Fig. S5B).

**Fig. 5:**
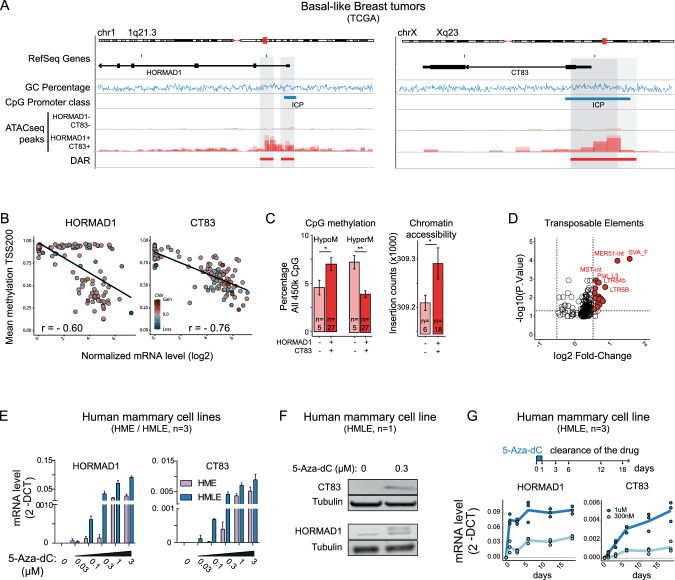
Activation of HORMAD1 and CT83 in tumors involves epigenetic alterations. **A** IGV representation of the HORMAD1 and CT83 genomic loci, with CpG density promoter classification according to the Weber/Schübeler criteria [89]. ATAC-seq data are from representative basal-like tumors (TCGA cohort). Differentially accessible regions (DAR) between these two groups of basal tumors were identified. **B** Inverse correlation between HORMAD1 and CT83 expression and the mean DNA methylation of their promoters (TSS ± 200 bp). Each dot represents a tumor and the color intensity indicates Copy Number Variation of the genomic locus. **C** Global epigenetic changes in basal-like breast tumors, according to HORMAD1 and CT83 expression status. Left: Total number of hypomethylated or hypermethylated CpG, expressed as percentage of all 450 K CpG with informative measure. Right: Chromatin accessibility, expressed as the number of accessible regions in ATAC-seq (mean ± SD). **D** VolcanoPlot of the differential expression of transposable elements, in basal-like tumors positive for HORMAD1 and CT83 expression vs. negative for both genes. **E** RT-qPCR analysis of HORMAD and CT83 expression in non-tumorigenic human mammary cell lines, in control condition or following a 48-h 5-Aza-dC treatment at the indicated concentrations. **F** Western Blot analysis of HORMAD1 and CT83 expression in non-tumorigenic human mammary cells, in control condition or following a 48-hour 5-Aza-dC treatment at 0.3 μM. **G** RT-qPCR analysis of HORMAD and CT83 expression at various time points, in the same cell line, after an initial perturbation with 0.3 or 1 μM 5-Aza-dC followed by a recovery period in drug-free medium.

As amplification seemed unlikely to explain the overexpression of HORMAD1 and/or CT83, we next examined epigenetic events. These genes lack CpG islands, but both have promoters with an intermediate CpG density (ICP) (Fig. 5A). These promoters overlap the ATAC-seq peaks which are present in HORMAD1/CT83-expressing basal-like breast tumors, but absent in non-expressing tumors (Fig. 5A). Consistently, we found histone marks associated with promoter activity (H3K27ac, H3K4me3) in sperm and in a basal-like cancer cell line positive for HORMAD1 and CT83 (MDA-MB-436) but not in the normal breast or negative breast cell lines (Fig. S5C, D). We next investigated the DNA methylation status of these promoters using the Illumina 450 K arrays available in TCGA and GEO. As shown in Fig. S5E, we found high levels of methylation on the HORMAD1 and CT83 promoters in normal breast samples (that do not express the genes) and low levels of methylation in the sperm samples (where the genes are on). The tumor data show a strong correlation between expression and promoter demethylation for CT83 (Fig. 5B). The correlation is present but less absolute for HORMAD1, as some tumors overexpress HORMAD1 without displaying demethylation.

Overall, tumors expressing HORMAD1 and CT83 show more permissive chromatin, with more hypomethylated regions compared to healthy tissue and more accessible chromatin regions (Fig. 5C). This hypomethylation is accompanied by the re-expression of many transposable elements, normally repressed by DNA methylation, in HORMAD1- and CT83- positive basal-like tumors compared to negative ones (Fig. 5D).

To understand whether demethylation is sufficient to induce HORMAD1 and CT83 expression, we used immortalized human mammary epithelial cells (HME and HMLE, [56]) treated in vitro with 5-aza-deoxycytidine (5-aza-dC). In the absence of treatment, we validated by RT-qPCR that these cell lines do not express HORMAD1 and CT83, in contrast to the triple-negative breast cancer line MDA-MB436 (Fig. S5F). The 5-aza-dC treatment induced both genes in a dose-dependent manner (Fig. 5E) and led to detectable protein expression (Fig. 5F). Importantly, the genes remained expressed even after removal of the drug (3 weeks time-course, Fig. 5G) and a recovery of global DNA methylation level (Fig. S5G), demonstrating a memory effect. This result is not general to C/T genes: other C/T genes known to be induced by 5-aza-dC (Fig. S5H) are indeed repressed within days to weeks after removing the drug.

From these data we conclude that promoter DNA methylation is associated with silencing of HORMAD1 and CT83 in the normal context, and that this mark is lost and replaced by active modifications such as H3K4me3 in cell lines and tumors that re-express the genes.

### HORMAD1 and CT83 act synergistically to increase stem-like cell proportions in vitro

**Fig. 6:**
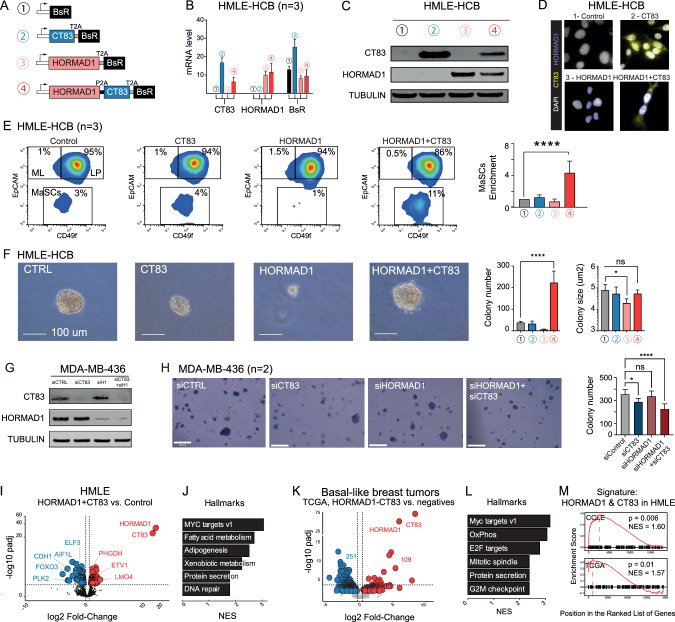
HORMAD1 & CT83 act synergistically to promote aggressive features in mammary cell lines. **A** Lentiviral vectors used to express HORMAD1 and/or CT83. P2A and T2A are self-cleaving peptides. BsR Blasticidin-resistance gene. **B** mRNA expression of HORMAD1, CT83, and the reporter BsR, assessed by RT-qPCR, in HMLE-derived cell lines. Data are represented as mean +/- SD (n = 3 independent experiments). **C** Western Blot analysis of HORMAD1 and CT83 expression in the indicated HMLE-derived cell lines. **D** Immunofluorescence staining of HORMAD1 and CT83 in HMLE-derived cell lines. **E** FACS analysis of EpCAM and CD49f cell-surface markers in HMLE-derived cell lines. Mature luminal cells (mL, EpCAM+CD49f−), luminal progenitor cells (Lp, EpCAM+CD49f+), mammary stem cells (MaSC, EpCAM-CD49flow) Top: One experiment representative of 3 independent experiments is shown. Bottom: summary of 3 independent experiments (Mean ± SD). **F** Soft agar experiment in HMLE-derived cell lines. One experiment representative of 3 independent experiments is shown. Bottom: Summary of 3 independent experiments (Mean ± SD). **G** Western blot validation of CT83 and HORMAD1 knockdown in MDA-MB-436 cells after siRNA transfection. **H** Soft agar experiment in MDA-MB-436 cells transfected with siRNA against either non-targeting control, CT83, HORMAD1 or both CT83 and HORMAD1 simultaneously. Figure is representative of 2 independent experiments. Bar graph represents a quantification summary of 2 independent experiments. **I** Volcano plot of the distribution of differentially expressed genes (*p*-value adjusted < 0.01) in HMLE cells expressing HORMAD1 and CT83, relative to control cells. Genes of particular interest are indicated. **J** Barplot displaying the top 6 pathways differentially activated in HORMAD1 and CT83-positive HMLE-derived cells compared to the control condition, from MSigDB c7_Hallmark annotation. X-axis corresponds to −log10 adjusted *q*-values. **K** Same as in G, in basal-like breast tumors (TCGA) according to HORMAD1 and CT83 expression status. **L** Same as in H, in basal-like breast tumors (TCGA) according to HORMAD1 and CT83 expression status. **M** GSEA analysis comparing the “HORMAD1 + CT83” signature detected in HMLE cells (88 genes, see **I**), to the ranked transcriptome of the double-positive cell lines in the CCLE dataset, or of double-positive tumors in the TCGA dataset.

HORMAD1/CT83 expression may be a bystander consequence of epigenetic instability, or it could have a positively selected function; in other words, the genes and their products could be either markers or actors of transformation. To investigate this question experimentally, we used the HMLE cells (human mammary epithelial cells expressing hTERT and large T/small T, Fig. S6A), which constitute a well-accepted model to study the genesis of basal-like tumors. We generated polycistronic lentiviral vectors to express HORMAD1 and/or CT83 and selected the infected cells with antibiotics (Fig. 6A). RNA and protein were expressed as expected by the different vectors (Fig. 6B, C). Importantly, these first experiments showed that expression of one gene was not sufficient to induce the other; this is consistent with our observation that the expression of HORMAD1 or CT83 alone is not equivalent to the expression of both genes. By immunofluorescence with the cognate antibodies, we confirmed the published nuclear localization of HORMAD1 and a perinuclear localization for CT83 that could correspond to the endoplasmic reticulum (Fig. 6D), these patterns matched those observed with GFP-tagged proteins (Fig. S6B). The expression of one protein (HORMAD1 or CT83) did not measurably affect the distribution of the other (Fig. 6D), suggesting that they function independently in different cellular compartments.

Because the simultaneous activation of HORMAD1 and CT83 is associated with a worse prognosis for basal-like tumors, we performed a series of functional experiments to examine the consequences of expressing one gene, the other, or both in concert. We first examined the growth rate and cell cycle distribution, and observed no significant difference between control cells and cells expressing HORMAD1 and/or CT83 (Fig. S6C, D). From this, we surmised that there is no positive selection for accelerated proliferation in cells expressing both HORMAD1 and CT83.

We then examined other cellular phenotypes relevant to tumorigenesis: cellular identity and stemness. HMLE cells grown in vitro maintain some of the heterogeneity and differentiation hierarchy of the mammary gland. FACS sorting using well-characterized markers (CD49f/EpCAM) showed, as expected, that the control cell population contained ~95% of Luminal Progenitor-like cells (LP), about 3% of mammary stem cell-like cells (MaSC), and about 1% cells resembling Mature Luminal (ML) cells (Fig. 6E). We then measured the proportion of each population after expression of HORMAD1, CT83, or both. Expression of HORMAD1 alone tended to decrease the percentage of MaSC, but this trend failed to reach statistical significance. Expression of CT83 alone failed to elicit detectable variations. In contrast, the co-expression of HORMAD1 and CT83 induced a highly significant, 4-fold increase of the MaSC compartment (Fig. 6E). This finding was confirmed using a different set of markers, CD24/CD44; there again we saw an increase of the CD44-positive, CD24-low compartment, corresponding to stem-like cells (Fig. S6E).

Considering that the co-expression of HORMAD1 and CT83 increased the proportion of stem-like cells in the HMLE population, we next wondered if this phenomenon would affect the transformed phenotype of these cells. To investigate this we performed a soft agar assay, which allows for estimating cell propensity for anchorage-independent growth: a hallmark of tumorigenesis. After three weeks of growing in a medium layered with agar, the colonies in each condition were photographed and counted. This quantification revealed that, compared to the control, the HMLE cells infected with both HORMAD1 and CT83 showed a significant increase in the number of colonies, more so than either CT83 or HORMAD1 alone (Fig. 6F). These results indicate that this co-expression impacts the HMLE cell’s ability to grow independently of cell anchorage. Interestingly, expressing HORMAD1 alone significantly reduced the size of the colonies, but this effect was absent when CT83 was co-expressed (Fig. 6F), again underscoring a possible cooperation between the two proteins.

To further corroborate this apparent synergistic effect of HORMAD1 and CT83 expression, we performed loss-of-function experiments in a triple-negative breast cancer line that endogenously expresses both genes, MDA-MB-436. Using siRNA targeting either HORMAD1, CT83, or a non-targeting control, we obtained efficient knockdown as judged by western blot (Fig. 6G). We note that removing HORMAD1 did not destabilize CT83, or vice versa (Fig. 6G). We then tested the tumorigenic properties of single or double knockdown cells using the soft agar assay (Fig. 6H). The results show that, compared to the control, the single CT83 knockdown had a mild negative effect on colony formation, the single HORMAD1 knockdown had no significant effect, and the double HORMAD1/CT83 knockdown had a negative effect significantly more marked than either single knockdown. These data support the notion that the co-expression of these genes leads to increased tumorigenicity.

Together, these functional assays demonstrate that the co-expression of HORMAD1 and CT83 synergistically results in increased cell stemness and transformation, overall resulting in a more tumorigenic phenotype. We then sought to further understand the molecular basis of this synergy by analyzing the transcriptional changes involved.

### Simultaneous HORMAD1 and CT83 expression triggers a transcriptional signature found in tumors

To better dissect the synergistic effect of HORMAD1 and CT83, we performed RNA-seq using HMLE cells expressing HORMAD1 and/or CT83. The expression of HORMAD1 or CT83 alone had no major impact on the transcriptome (Fig. S6F). Conversely, the co-expression of the two genes induced a specific gene signature, with 88 differentially expressed genes, 49 down and 39 up (Fig. 6I, RNA-seq data available at GSE234475, reviewer token mfojwmwaplchjyp). Among these, we found genes known to be associated with basal breast cancer and/or mammary stem cells, such as the upregulated genes ETV1 - a nuclear effector of Ras-Kinase signaling involved in cell proliferation and differentiation - [57, 58], PGHDH -an enzyme involved in glycolysis, upregulated in basal-like breast cancers [59]- and LMO4 -an inhibitor of mammary epithelial cell differentiation [60, 61]-, or the repressed tumor suppressor genes ELF3 -which increases apoptosis in non-transformed mammary epithelial cells [62, 63] -, FOXO3 - an unconventional tumor suppressor gene with pro-apoptotic function [64], and PLK2 -another tumor suppressor gene [65] (repressed). We then searched for biological functions associated with this “HORMAD1 + CT83” signature by performing Gene Ontology (GO) analyses on curated signatures or general pathways. We found significant associations of this signature with several breast cancer-related pathways and with pathways associated with epithelial-to-mesenchymal transition and specific signaling pathways of the mammary gland that may act on cell differentiation/homeostasis (Fig. 6J). We verified these RNA-seq results using RT-qPCR on selected genes (Fig. S6H, I) and further investigated the upregulation of well-known master regulators of the EMT program; amongst them, TWIST1 and ZEB1 are upregulated in HMLE overexpressing HORMAD1 and CT83 (Fig. S6J). Activation of the EMT, even partial [66, 67], as well as the stimulation of pathways related to stemness and tumorigenesis, could contribute to the increase in the proportion of stem cells observed as well as the growth properties in the absence of anchoring.

We subsequently asked whether the “HORMAD1 + CT83” signature, as seen in vitro, is germane to the transcriptional profile of cancer cell lines and basal tumors expressing both genes. For this, we started by performing differential gene expression analysis on the double-positive vs. double-negative basal cell lines within the CCLE. This yielded a transcriptomic profile including 440 differentially regulated genes (Fig. S6K). Next, we carried out a similar analysis on the basal-like tumors in the TCGA. The transcriptomic profile of HORMAD1/CT83 double-positive tumors was different from that of double-negative tumors and included 560 differentially expressed genes (Fig. 6K). Upregulated pathways overlapped some of the pathways identified in HMLE cells (ie. Myc Targets v1) and were mostly involved in cell proliferation (Fig. 6L). A GSEA analysis revealed that the “HORMAD1 + CT83” signature detected in HMLE cells was significantly correlated to the transcriptome profile of double-positive cancer cell lines (Fig. 6M, upper panel) and with the transcriptome of double-positive tumors (Fig. 6M, lower panel).

In summary, these various bioinformatic, transcriptional and functional analyses results establish that the co-expression of HORMAD1 and CT83 in breast cells has effects different from the expression of either gene alone. Phenotypic analyses reveal this co-expression leads to increased tumorigenic properties in HMLE cells, while the RNA-seq results showed this co-expression is sufficient to induce a transcriptional program that resembles that of basal-like breast tumors. In total, these results illuminate the potential for HORMAD1 and CT83 use as biomarkers for basal-like breast cancers by showing both their specificity for the subtype as well as the functional consequences of their co-activation.

## Discussion

### A new approach identifies cancer/testis genes expressed in different breast tumor subtypes

Cancer/Testis genes hold promise as markers, actors, and targets in cancer. Here we implement a new bioinformatic approach to identify the Cancer/Testis genes that are overexpressed in breast cancer. This approach has the advantage of being rigorous and calculation-efficient, immediately usable for any tumor type, and easily adaptable to seek other types of genes misexpressed in tumors. It complements previous approaches based on expression thresholds [12] or vector colinearity [30], yielding results that either approach alone would not have produced (Fig. 1B). Combined with machine learning on large breast cancer cohorts, this method reveals new markers specific to breast cancer subtypes. Most were previously unknown, and some were associated with prognosis and response to treatment, rendering them potentially valuable markers. Future investigations could also examine whether these markers actively participate in the transformation process. Our examination of early-stage tumors reveals that the pattern of Cancer/Testis gene expression is determined early on, and could even be inherited from rare abnormal cells existing in the healthy mammary gland, which has interesting practical and conceptual implications.

With our approach we identify two genes of interest, HORMAD1 and CT83, which show expression in most basal tumors, but few other tumors of different subtypes. HORMAD1 is expressed in preleptotene spermatocytes [38] and is required to promote non-conservative recombination events in meiosis and the resulting formation of the synaptonemal complex [68–70]. CT83 (also known as CXorf61 or KK-LC-1) encodes a small protein (113 AA) of unknown function, normally expressed in mature sperm [71]. While both genes have been previously linked to basal tumors [34, 36, 72–76], our work goes further and brings a number of novel findings : 1) we rigorously prove that the genes are the two strongest predictors of a tumor being basal in independent cohorts, 2) we have precisely described their expression pattern before the appearance of a tumor and during its evolution, 3) we demonstrate that they have a synergistic effect on breast cells.

Three important questions remain open and will be discussed briefly in the following paragraphs: what is the order of events leading to HORMAD1/CT83 induction in basal tumors? What are the mechanistic bases for their induction? And how do the two genes exert their synergistic effect?

### Order of events

Approximately 90% of basal tumors in the TCGA cohort express HORMAD1 or CT83, and about 60% express both. There are two non-exclusive interpretations for these high proportions.

First, the induction of the genes could be an early event that occurs in most early lesions and is maintained as the tumor progresses. In principle, this deregulation could even occur earlier than the main transforming event, such as the well-known phenomenon of oncogene activation in healthy tissues [77, 78]. It could be that HORMAD1/CT83 induction reflects a disturbed epigenetic landscape in rare tumor-initiating cells, which could itself increase the probability of cellular transformation [5, 79, 80]. In this scenario, HORMAD1 and CT83 themselves could be markers of the early epigenetic instability, or they could actively participate in the ensuing transformation. One piece of data supporting this “induction before transformation” hypothesis is that a few rare cells in the healthy breast already express CT83 and/or HORMAD1. Some of those aberrant cells might eventually be amenable to enter the basal-like transformation path.

In the alternative possibility, the expression of HORMAD1 and CT83 occurs after transformation, equipping basal tumor cells with a selective advantage. These genes have only been studied individually so far, however compelling evidence suggests that HORMAD1 overexpression impairs homologous recombination and increases genetic instability in basal breast tumor cells, possibly speeding up tumor evolution [34]. HORMAD1 overexpression is also detected in lung tumors [81] but, paradoxically, it seems to increase the robustness of homologous recombination in these tumors, making them more resistant to DNA-damaging chemotherapy. These divergences may mean that HORMAD1 has context-dependent functions, for instance, in the presence or absence of other actors such as CT83.

### Mechanism of induction

Though basal tumors are genetically unstable, we rule out gene amplification as the primary mechanism of HORMAD1/CT83 induction. Instead, we show that DNA methylation is a barrier to HORMAD1/CT83 activation, consistent with previously published reports [81, 82]. Importantly, we find that once the genes have been induced by a 5-aza-deoxycytidine treatment, they remain active even when 5-aza-dC has been removed. In other words, they switch to a stable “On” state, rendering them excellent markers of past epigenetic disturbances.

Further investigations will be required to elucidate the initial event(s) that lead to the derepression of HORMAD1/CT83 at some point during the history of most basal tumors. It could be a stochastic phenomenon occurring before or after transformation; alternatively it could be a directed event triggered by the transforming pathway(s). While many Cancer/Testis genes are repressed by DNA methylation, HORMAD1 and CT83 are highly specific in their association with basal tumors. Therefore, they could be specifically induced in this tumor type, specifically selected for, or both.

### Synergy between HORMAD1 and CT83

Using in vitro models of breast cells and gain-of-function tools, we show that the joint expression of HORMAD1 and CT83 has transcriptional and phenotypic consequences that are not observed when either gene is expressed in isolation.

In breast cells, joint HORMAD1/CT83 expression increases the proportion of stem-like cells. This correlates with the induction of a transcriptional signature that is also found in double-positive basal tumors. Therefore, the combined expression of HORMAD1 and CT83 is sufficient to increase stem-like properties, and to kick-start a transcriptional program observed in the basal tumors that have the poorest prognosis. This finding suggests that HORMAD1 and CT83 are not merely markers but also actors of basal breast tumorigenesis.

We find that neither protein is sufficient to turn on the production of the other, and that neither protein detectably affects the amount or localization of the other. Discovering the mechanistic underpinnings of the cooperation between HORMAD1 and CT83 remains an open question for future investigations. A related question is how to therapeutically target these proteins and/or their collaborative function.

### Limits and perspectives

We note that our analysis has a number of possible limitations. One is that we used pre-existing lists of Cancer/Testis genes; any gene not detected in these previous publications has not been considered in our work. Another has to do with sensitivity: if certain genes are expressed only in a small number of tumors, then the smoothing we performed in the initial step of our analysis may have made them undetectable. Our sample size was large, with more than 1000 tumors, but certain rare subtypes (such as normal-like tumors, only represented by 40 data points) may benefit from a more focused approach. Also, we focused on one specific type of genes misexpressed in tumors: the Cancer/Testis genes. However, other tissue-specific genes ectopically expressed in breast tumors can be a rich source of markers and may be involved in the transformation process. These genes can be easily recovered from our dataset and may deserve further investigations in the future.

In spite of the limitations mentioned above, the current work brings new conceptual insight into the role of Cancer/Testis genes in breast cancer, showing that their abnormal activation is a rare phenomenon in healthy tissues but very frequent in tumor cells, occurring very early during oncogenesis, and possibly having a synergistic effect. In practical terms, as already underlined by other investigators [33, 83–85], the genes we have studied represent potential targets for immunotherapy. We show, in addition, that their epigenetic activation seems irreversible, and that they could constitute ideal witnesses of past episodes of epigenetic instability. This may help better understand the role of epigenetic instability in breast tumors, and its mechanistic connection to cellular transformation.

## Materials and methods

### Wet biology

#### Cell culture

Human mammary cell lines, derived from normal mammary tissue, were obtained from collections developed and generously given by the laboratories of Christophe Ginestier (CRCM) and Raphaël Margueron (Institut Curie). Cancer cell lines (MDA-MB-436, HEK293T) were obtained from ATCC or generously given by the laboratory of Marc-Henri Stern (Institut Curie).

HME and HMLE cells were grown in DMEM:F12 medium supplemented with 10% FBS, 1% penicillin/streptomycin, Non-essential Amino Acids (LifeTechnology 11140-035) 1%, Insulin Humalog (Lily) 10 μg/ml, Hydrocortisone (Serb) 0.5 μg/ml, EGF (ThermoFisher PHG0311) 10 ng/ml. HEK293T and MDA-MB-436 were grown in DMEM medium supplemented with 10% FBS, 1% penicillin/streptomycin.

Cells were incubated in a humidified atmosphere at 37 °C under 5% CO_2_. All experiments were performed on subconfluent cells in the exponential phase of growth.

#### Transfection of cells with siRNA

Cells were transfected with siRNA using Lipofectamine RNAimax according to the manufacturer’s protocol. For knockdown studies, cells were seeded in 6-well plates at a density of 2.5 × 10^5^ and transfected with siRNA produced by Thermofisher for either a non-targeting negative control (#4390843), or targeting CT83 (#4392420, s47508), HORMAD1 (#4392420, s38456) or both simultaneously. Knockdown efficiency was confirmed with RT-qPCR and western blot.

#### Soft Agar assay

For soft agar experiments, a base layer of 1% agarose diluted in 2× DMEM + 10% FBS was first laid in 6-well tissue culture plates and left to solidify at room temperature. Cells were then seeded at a 2·10^4^ density in a second layer of 0.7% agarose mixed with 2× DMEM and allowed to solidify. Wells were then supplemented with a final feeder layer of 1X DMEM medium which was changed every 3 days. Cells are left to grow between 2–3 weeks, then stained with 0.005% Crystal Violet and quantified using ImageJ, statistical significance was determined with an ANOVA test. The experiment was performed twice with 7 technical replicates per experiment for reproducibility.

#### Treatment of cells with 5-aza-dC

Treatment with 5-Aza-dC was performed as described previously [13]. Briefly, for dose-response experiments, cells were seeded at a density of 1. 10^4^ cells in a 6-well tissue culture plate. When cells became firmly adherent to plastic, the medium was replaced with fresh medium containing the appropriate concentration of 5-Aza-dC, every 24 h for 2 days (two pulses). When cells became firmly adherent to plastic (T0), the medium was replaced with fresh medium containing 1 μM or 300 nM of 5-Aza-dC for 24 h (one pulse). At the end of the treatment, the medium was replaced with fresh culture medium without 5-Aza-dC, and cells were cultured for an additional 2 weeks in subconfluent condition with regular passages. At the end of the treatment and at the appropriate time points, cells were used for molecular assays. Control cultures were treated under similar experimental conditions in the absence of 5-Aza-dC.

#### Generation of the HORMAD1 and/or CT83 mammary cell lines

The maximal reporter cassette comprised HORMAD1-P2A-CT83-T2A-Blasti^R^ (Synthesized by GenScript). The three proteins expressed by the cassette were separated from each other by self-cleaving 2A peptides (P2A, T2A). This cassette was cloned in a lentiviral backbone from ORIGENE (derived from PS100071), under the control of the constitutive CMV promoter. The control plasmid (Blasti^R^) and the two other plasmids (HORMAD1 -T2A-Blasti^R^ and CT83-T2A-Blasti^R^) were generated by enzymatic digestion; all the plasmids were grown and prepared individually. The sequences were validated by sequencing. Lentiviruses were generated and used for transduction. Production of lentiviral particles was performed by calcium-phosphate transfection of HEK293T with psPAX2 and pMD2.G plasmids, in a BSL3 tissue culture facility. HME or HMLE cells were seeded into 12-well plates, infected, and selected with blasticidin (5 μg/ml) for 15 days.

#### Western blotting

Cells were harvested and lysed in RIPA buffer (Sigma) with protease inhibitor cocktail (Thermo Fisher Scientific), sonicated with a series of 30 s ON/30 s OFF for 5 min on a Bioruptor (Diagenode), and centrifuged at 16,000 × *g* for 5 min at 4 °C. The supernatant was collected and quantified by BCA assay (Thermo Fisher Scientific). Thirty microgram protein extract per sample was mixed with NuPage 4× LDS Sample Buffer and 10X Sample Reducing Agent (Thermo Fisher Scientific) and denatured at 95 °C for 5 min. Samples were resolved on a pre-cast SDS-PAGE 4–12% gradient gel (Thermo Fisher Scientific) with 120 V electrophoresis for 90 min and blotted onto a nitrocellulose membrane (Millipore). The membrane was blocked with 5% fat-free milk/PBST at RT for 1 h, then incubated overnight at 4 °C with appropriate primary antibodies. After three washes with PBS/0.1% Tween20, the membranes were incubated with the cognate fluorescent secondary antibodies and revealed in the LI-COR Odyssey imaging system. The following antibodies were used in this study: α-HORMAD1 (dilution 1:1000, reference HPA037850), α-CT83 (dilution 1:1000, reference HPA004773), α-Tubulin (dilution 1:10 000, reference Abcam ab7291).

#### Quantitative real-time PCR

RNA was extracted using Tri reagent according to the manufacturer’s recommendations. One microgram of total RNA was reverse transcribed using SuperScript IV Reverse Transcriptase (Thermo Fisher Scientific) and Oligo dT primers (Promega). qPCR was performed using Power SYBR Green (Applied Biosystems) on a Viia 7 Real-Time PCR System (Life Tech). *TBP* and *PGK1* genes were used for normalization of expression values. Primer sequences are available in Supplementary Table S4.

#### Immunohistochemistry

Paraffin-embeded tissue blocks from a 99 cohort of breast cancer patients, obtained at the time of initial diagnosis, were retrieved from the archives of the Department of Diagnostic and Theranostic Medicine, Curie Institute. Sections of 3 μm in thickness were cut with a microtome. Tissue sections were deparaffinized and rehydrated through a series of xylene and ethanol washes. Immunohistochemistry stainings were performed using HORMAD1 (dilution 1/200) rabbit antibody reference PA5-58138 Thermofisher) and CT83 (dilution 1/300, rabbit antibody reference HPA004773, Invitrogen). Briefly, key figures included: (i) antigen retrieval in 0.1 mol/L citrate buffer, pH 9 (BioCare, Pacheco, CA, USA) in a pressure cooker (4 min); (ii) blocking of endogenous peroxidase activity by immersing sections in 3% hydrogen peroxide in methanol for 15 min and subsequently rinsing them in water and PBS; (iii) incubation with primary antibodies against the targeted antigen; (iv) immunodetection with a biotin-conjugated secondary antibody formulation that recognizes rabbit and mouse immunoglobulins, followed by peroxidase-labeled streptavidin and linking with a rabbit biotinylated antibody against mouse immunoglobulin G and (v) chromogenic revelation with DAB and counterstaining with Mayer’s hematoxylin. The specificity of the HORMAD1 and CT83 antibodies were confirmed via the same protocol on paraffin-embedded human tissue sections of healthy testis tissue. A semi quantitative histological score (HScore = intensity × frequency) was used for interpretation (0 = negative staining, 1 = weak staining, 2 = moderate staining and 3 = strong staining).

#### Immunofluorescence

Cells were grown on glass coverslips, fixed with 4% paraformaldehyde (PFA) for 10 minutes, and then permeabilized with 0.5% Triton. The glass coverslips were then blocked with 1% bovine serum albumin in phosphate buffer saline for 1 h, before applying primary antibody for 1 h. After this incubation, secondary antibody was applied for 45 min, before washed and applied Hoechst stain (1:20,000; Sigma #33258). The following antibodies were used: Anti-HORMAD1 (HPA037850; 1/3000), Anti-CT83 (C; 1/200); Donkey anti-Rabbit Alexa Fluor 488 (1/2000), Donkey anti-Mouse Alexa Fluor 594 (1/200).

#### Flow cytometry

Freshly dissociated cells were stained with APC-conjugated EpCAM (dilution 1:100, Miltenyi clone HEA-125) and PerCP-Cy5.5-conjugated CD49f (dilution 1:10, BD clone GoH3); or APC-conjugated CD44 (dilution 1:10, BD clone G44-CD26) and PerCP-Cy5.5-conjugated CD24 (dilution 1:10, BD clone ML5); with live/dead Violet (dilution 1:1000, ThermoFisher) for cell viability, in HBSS (Gibco) with 2% FBS and incubated at room temperature for 20 min, followed by washing in HBSS with 2% FBS and re-suspended in HBSS/FBS 2%. Analysis was performed by using a CyAn (Beckman Coulter) flow cytometer. Thresholds on fluorescence signal intensity (subtracting background fluorescence from the appropriate isotype control antibodies) were used to determine the proportion of cell populations. Data were analyzed with FlowJo software.

### Bioinformatics

#### Public data sets used in this study

We used previously published gene lists to define testis-specific genes, tumor suppressor genes and oncogenes. We also used multiple public datasets involving both normal and tumor tissues to evaluate C/T gene expression. Detailed information of these databases was listed in the Supplementary Table 5.

#### Development of the Cancer-Gene Marker Detection pipeline

Briefly, we computed the Kernel’s density estimation for each gene expression pattern in healthy mammary gland and in breast cancer cohorts, respectively. We then analyzed density profile variations using the derivative of the density functions and classify genes as unimodal or multimodal in normal mammary tissues and breast cancer samples. For each gene, we compared the mean expression values in normal and cancer samples using the nonparametric Wilcoxon’s test, with a significance level of 0.01. We classify genes according to these parameters, as described in Fig. S1A-C. All the detailed scripts are available on GitHub (https://github.com/MartheLaisne/CTA_BreastCancers).

#### Identification of genes with abnormal breast cancer expression pattern using transcriptomic TCGA analysis

TCGA gene count datasets for breast normal and cancer samples were downloaded using TCGAbiolinks [86]. Expressions were normalized with DESeq2 (Love MI, Huber W, Anders S, v.1.22.2). Abnormally expressed genes were defined based on the kernel density estimation of gene expression in normal and cancer samples, respectively, and their derivatives, as followed: any gene with no expression in normal mammary gland samples (unimodal distribution of gene expression values, centered on zero) but a significant expression in some cancer samples (multimodal distribution of gene expression values in breast cancer samples, with mode2 > 0). All the detailed scripts are available on GitHub.

#### RandomForest

We used randomForest (Breiman et al. v4.16.14) and Caret (Kuhn et al. v6.0) packages to assess the predictive potential of 139 selected C/T gene expressions for breast cancer subtypes, aiming to identify the most informative genes. Our analysis involved two independent datasets, namely TCGA Breast and [37]. For the two datasets we employed the same strategy: w, we randomly partitioned the samples into a training set (comprising 75% of the full dataset) and a test set (the remaining 25%).

To optimize the hyperparameters of the random forest model, we employed default parameters from the Caret package, utilizing the train and trainControl functions. This optimization process incorporated threefold cross-validations and was repeated across 10 resampling iterations. In both cases, we ensured that the chosen number of trees (500 estimators) allowed the model to convergence.

The best-performing model, determined by the highest accuracy value, was selected from the random forest trial. Subsequently, we assessed this model on the test set (comprising the remaining 25% of samples) by constructing a confusion matrix comparing the model predictions with the true annotations for these samples. Notably, specificity and sensitivity were computed using a ‘one versus all’ approach, comparing each factor level to the remaining levels. The ranking of the 139 C/T genes was established according to their predictive values in the multiclass classification, with 5 classes for TCGA dataset (Basal, Her2, LumA, LumB and Normal-like).

#### Validation of the Testis-specific expression pattern for the 139 selected C/T genes

Expression values for GTEx [87] dataset was obtained directly from the project webpage as TPM values, and the median expression values by tissue were calculated. We extracted expression values for the 139 selected TS genes, and we performed an unsupervised clustering (Euclidean distance and complete method) of the genes and the samples based on these values. Detailed script is on GitHub.

#### Analysis of the INVADE dataset

The INVADE cohort is a retrospective series of 55 patients, who have been treated at Institut Curie between 1992 and 2014, and underwent surgery for a breast carcinoma prior to any treatment. This cohort includes 18 pure DCIS cases, 17 microinvasive (MI-DCIS) cases (DCIS lesions with invasive foci of maximum 1 mm) and 20 primary IBC cases. Informed patient consents for the use of tissues for research purposes were collected, and ethical approval from the Institutional Review Board (Institut Curie breast cancer study group) was obtained for the use of all specimens.

Frozen samples were processed for RNA extraction using kit (miRNeasy Mini Kit, Qiagen #217004) following the manufacturer’s instructions. RNA integrity and quality were analyzed using Agilent 4200 TapeStation system. The library was prepared following the protocol of the Illumina® TruSeq Stranded mRNA kit according to the supplier’s recommendations. Briefly, the key steps of this protocol were successively, starting from 1 µg of total RNA: purification of PolyA (containing mRNA molecules) using magnetic beads attached to poly-T oligonucleotides, fragmentation using divalent cations at high temperature to obtain fragments of approximately 300 bp, cDNA synthesis, and finally ligation of Illumina adapters and amplification of the cDNA library by PCR. Sequencing was then performed on the Illumina HiSeq2500 sequencer (75-bp paired end). Image analysis and base-calling were performed using Illumina Real-Time Analysis (RTA 2.1.3) with default settings. TopHat2 (v2.0.10) was used to align the raw RNAseq data on the human genome (hg19) and on a transcriptome from the refSeq annotations (April 2015 version) with the following parameters: bowtie2 (v2. 1. 0) [88] using the sensitive and fr-firsttrand parameters for the library type (strand specific protocol), allowing up to 2 mismatches in the seed of 25 bp and a gap of up to 10 bp in alignment, an intron size between 30 bp and 700 kbp, with a mean insert size between read pairs of 155 bp with a standard deviation of 80 bp. Raw counts were then calculated by reconstructed transcripts (26,093 genes), using Cufflinks toolkit (v2.2.1), using default parameters and stranded mode. Then, raw counts were normalized using DESeq2 (Love MI, Huber W, Anders S, v.1.22.2). Because there are no normal tissues in this dataset, another strategy was used to define the threshold for abnormal C/T gene activation: we used the bimodality of the expression values distribution to define a background level. Any expression value below this threshold was considered as noise, and the gene as repressed. The top 20 CT genes based on random forest analyzes were used to perform an unsupervised hierarchical clustering (binary distance and Ward.D2 method) of the 55 tumor samples. Detailed script is on GitHub.

#### Analysis of normal breast microarray

Data were downloaded at https://www.ebi.ac.uk/arrayexpress/experiments/E-MTAB-4145. The raw CEL data were normalized using the following packages: affy (v1.60.0), ArrayExpress (v1.42.0) for annotation and data importation; oligo (v1.45.0), arrayQualityMetric (v3.38.0) for quality control and preprocessing; limma (v3.38.3) for analysis and statistics.

#### scRNA-seq of normal breast cells

Briefly, data from the two datasets were downloaded (GSE113197, GSE161529) and separately analyzed using the Seurat (v3.1.4) package. We filtered low quality cells by (i) few expressed genes, (iii) abnormally high number of expressed genes and (iii) high mitochondrial gene expression. After these steps, there remained 23,007 cells for the GSE113197 set, and 79,097 cells for the GSE161529 set.

UMAP was constructed based on the minimal number of significant PCs from PCA. Cell identities were assigned based on the expression of lineage markers. Detailed scripts are on GitHub.

#### scRNAseq of triple-negative breast tumors

FASTQ read pairs were aligned to the human reference genome (build gencode v29) using STAR (v2.7.5c) and default single-pass parameters. Uniquely aligned reads were kept for downstream analysis using Samtools view (v1.10) and parameters: -q 10 -b –o, and counted with htseq (--stranded=yes –type=exon). Data were analyzed using Seurat (v3.1.4). As for Healthy mammary scRNA-seq analysis, we identified low quality cells by (i) few expressed genes, (iii) abnormally high number of expressed genes and (iii) high mitochondrial gene expression. Cell identities were determined using the same procedure as for the healthy mammary scRNA-seq data. We also used Lehman signature to assign each cancer cell to a lehman subtype, as described in the original publication (code source: https://github.com/Michorlab/tnbc_scrnaseq).

#### Differential gene expression analysis in TCGA basal-like samples

We classified tumors in 4 different groups as described, reflecting their expression levels of both HORMAD1 and CT83. Then, we downloaded HTseq-counts data for basal-like breast tumors only and we performed a differential expression analysis using the R package *DESeq2*, with the HORMAD1 & CT83 label as factor of interest. Differentially expressed genes were defined with p-adjusted < 0.05 and absolute value for the fold-change > 1.5.

#### Differential peak intensity analysis in TCGA basal-like samples

Both raw counts ATAC-seq data and gene expression data from TCGA were accessed (2020 accession) through either the Genomic Data Commons (GDC) using the GDC Data Transfer Tool Client or the data transfer tool TCGAbiolinks [86]. Individual patient files were assembled using in-house scripts in an R computing environment. Preprocessing consisted of patient and gene matching between data types, log transformation of gene expression data, and classification of the ATAC-seq samples regarding their HORMAD1/CT83 expression status, defined in the previous section. For differential analysis, we used basal-like tumors from ATAC-seq data (*n* = 30). Differential peak intensities were found using *DESeq2*. Differentially open regions were defined with p-adjusted < 0.01 and absolute value for the fold-change > 2.

#### CpG promoter class identification

Promoters were according to the hg38 version of the human genome, as described in the original article [89]. Briefly, promoters were classified in three categories to distinguish strong CpG islands, weak CpG islands and sequences with no local enrichment of CpGs. We determined the GC content and the ratio of observed versus expected CpG dinucleotides in sliding 500-bp windows with 5-bp offset. The CpG ratio was calculated using the following formula: (number of CpGs × number of bp)/(number of Cs × number of Gs). The three categories of promoters were determined as follows: HCPs (high-CpG promoters) contain a 500-bp area with CpG ratio above 0.75 and GC content above 55%; LCPs (low-CpG promoters) do not contain a 500-bp area with a CpG ratio above 0.48; and ICPs (intermediate CpG promoters) are neither HCPs nor LCPs.

#### Correlation DNA methylation data and expression data for TCGA samples

Both DNA methylation data, Copy Number Variations (CNV) data and gene expression data from TCGA were accessed (2020 accession) through either the Genomic Data Commons (GDC) using the GDC Data Transfer Tool Client or the data transfer tool TCGAbiolinks [86]. Individual patient files were assembled using in-house scripts in an R computing environment. Preprocessing consisted of patient and gene matching between data types and log transformation of gene expression data. The methylation data in this study were acquired by the Illumina 450 K array, which interrogates more than 450,000 methylation sites on the Illumina chip. The data for this study contained information of 485,578 CpG sites. The CNV data were acquired by the Affymetrix SNP 6.0 array numeric CNV values derived from GISTIC2.

Correlation analysis was performed using Pearson’s correlation. The correlation was performed between methylation beta values (respectively between CNV values) and log-base-2-transformed gene expression data with a *p*-value threshold of ⩽.05. All statistical tests used standard R functions.

#### Expression correlation between adjacent genes in the TCGA data

Correlation analysis was performed using Pearson’s correlation. The correlation was performed between the two log2 normalized adjacent genes expression values. All statistical tests used standard R functions.

#### RNA-sequencing: library preparation for transcriptome sequencing

A total amount of 1 μg total RNA per sample was used as input material for the RNA sample preparations. RNA samples were spiked with ERCC RNA Spike-In Mix (Thermo Fisher Scientific). Sequencing libraries were generated using NEBNext UltraTM RNA Library Prep Kit for Illumina (NEB) following the manufacturer’s recommendations. Briefly, mRNA was purified from total RNA using poly-T oligo-attached magnetic beads. Fragmentation was carried out using divalent cations under elevated temperature in NEBNext First-Strand Synthesis Reaction Buffer (5×). First-strand cDNA was synthesized using a random hexamer primer and M-MuLV Reverse Transcriptase (RNase H-). Second strand cDNA synthesis was subsequently performed using DNA Polymerase I and RNase H. In the reaction buffer, dNTPs with dTTP were replaced by dUTP. The remaining overhangs were converted into blunt ends via exonuclease/polymerase activities. After adenylation of 3′ ends of DNA fragments, NEBNext Adaptor with hairpin loop structure was ligated to prepare for hybridization. To select cDNA fragments of preferentially 250–300 bp in length, the library fragments were purified with the AMPure XP system (Beckman Coulter). Then 3 μl USER Enzyme (NEB) was used with size-selected, adapter-ligated cDNA at 37 °C for 15 min followed by 5 min at 95 °C before PCR. Then PCR was performed with Phusion High-Fidelity DNA polymerase, Universal PCR primers, and Index (X) Primer. At last, products were purified (AMPure XP system) and library quality was assessed on the Agilent Bioanalyzer 2100 system.

#### RNA-sequencing: read alignment

FASTQ reads were trimmed using Trimmomatic [90] (v0.39) and parameters: ILLUMINACLIP:adapters.fa:2:30:10 SLIDINGWINDOW:4:20 MINLEN:36. Read pairs that survived trimming were aligned to the human reference genome (build hg38) using STAR [91] (v2.7.5c) and default single-pass parameters. PCR duplicate read alignments were flagged using Picard-tools (2019) MarkDuplicates (v2.23.4). Uniquely aligned, non-PCR-duplicate reads were kept for downstream analysis using Samtools [92] view (v1.10) and parameters: -q 255 -F 1540. Gene expression values were calculated over the hg19 NCBI RefSeq Genes annotation using VisRseq [93] (v0.9.12) and normalized per million aligned reads per transcript length in kilobases (RPKM). Bigwig files were generated using deeptools [94] bamCoverage (v3.3.0) using counts per million (CPM) normalization and visualized in IGV [95] (v2.8.9).

#### RNA-seq: differential expression, PCA plots, and heatmaps

All the analysis and figures were generated using custom scripts and R version 3.5.2. Scripts are available on Github (https://github.com/MartheLaisne/CTA_BreastCancers/). Raw and normalized data are available here: GSE234475. The reviewer token is mfojwmwaplchjyp.

#### Gene set enrichment analysis (GSEA)

Gene set enrichment analysis was performed using GSEA [96, 97] (v4.1.0), msigdbr and fgsea package and default parameters (1000 permutations, permutation type = gene_set. Selected significant terms from Hallmark gene sets (*n* = 50), KEGG gene set (*n* = 186), GO biological functions (*n* = 1001) and Curated Breast Pathways (*n* = 169) were displayed. Curated breast gene set is available in Supplementary Table 3.

### Supplementary information

Supplementary Figures

Supplementary Table 1

Supplementary Table 2

Supplementary Table 3

Supplementary Table 4

Supplementary Table 5

## Data Availability

The RNA-seq data generated in the course of this work is publically available under the GEO reference GSE234475.
