# Supplementary material for: A novel bioinformatic approach reveals cooperation between Cancer/Testis genes in basal-like breast tumors
Source: Oncogene. 2024 Mar 11;43(18):1369–85. doi: 10.1038/s41388-024-03002-7 (PMC11065691; doi:10.1038/s41388-024-03002-7)
Supplement: Supplementary file 1 — Supplementary Figures [file 41388_2024_3002_MOESM1_ESM.pdf]

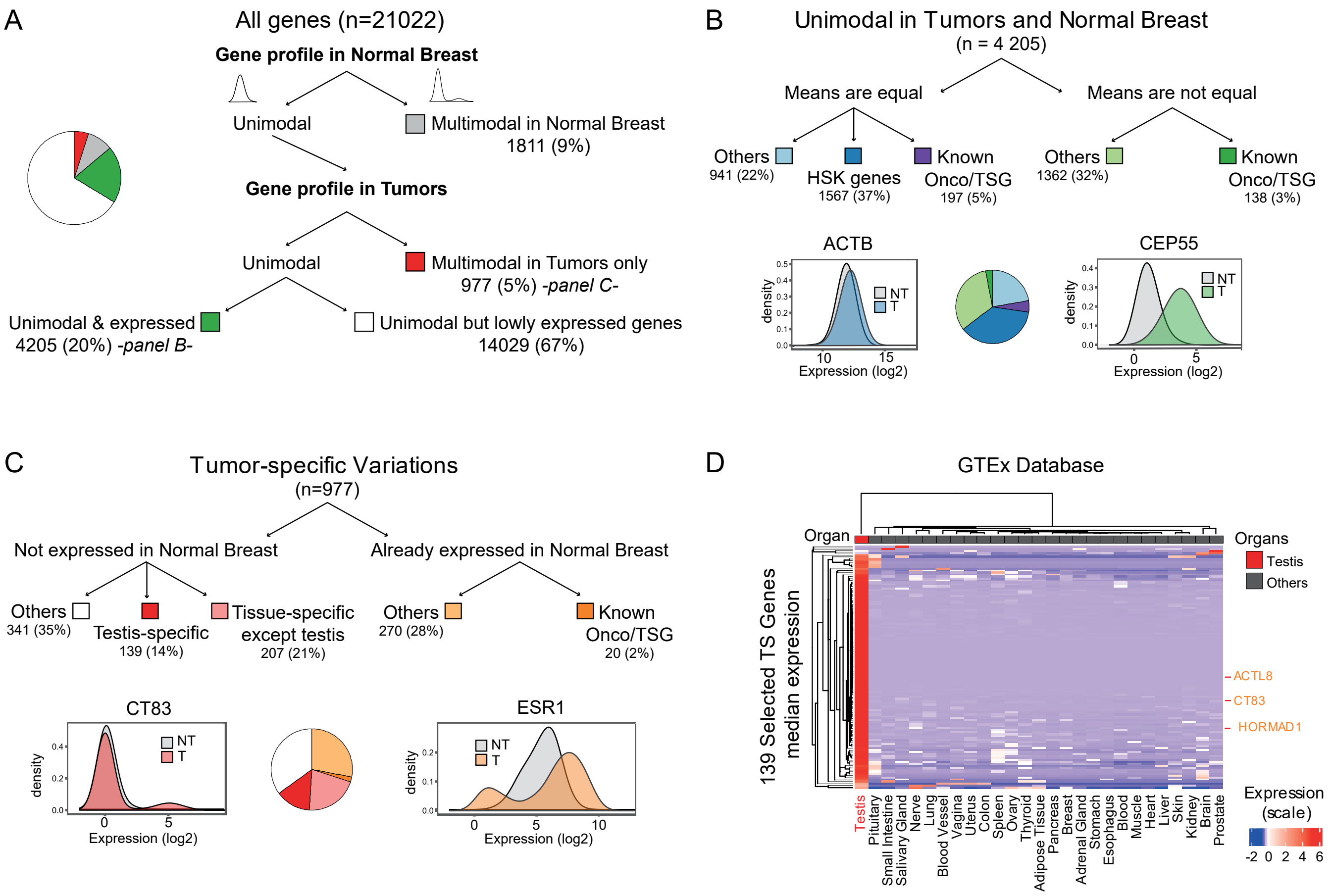

Figure S1: Further uses and validation of our novel bioinformatic screening approach

A. Classification of all genes according to our parameters: we were interested in genes with a homogeneous expression in Normal Breast (NB); ie. with a unimodal profile in Normal Breast. These genes were subsequently divided according to their expression pattern in breast tumors, with two situations of specific interest: genes also homogeneously expressed in breast tumors (panel B) and genes that are overexpressed or repressed in a subset of breast tumors (panel C).

B. Refinement of the characterization of genes homogeneously expressed both in Normal Breast and in breast tumors: when means were significantly different between Normal Breast and tumors, these genes could be used as tumor markers. Some of these genes are known as overexpressed oncogenes or repressed tumor suppressor genes; a significant portion (1362 genes) have no currently known role in cancer but could play a role in breast tumor development. HSK: housekeeping gene; TSG: Tumor Suppressor Gene. Onco: oncogene.

C. Refinement of the characterization for tumor-specific variables genes: approximately 70% of them are repressed in Normal Breast and abnormally activated in breast tumors; amongst these genes are known tissue-specific genes (including testis-specific genes). The remaining 30% are overexpressed or repressed genes in some breast tumors, including known subtype-specific oncogenes like ESR1 and other genes that could be used as a marker of specific tumor subgroups.

D. Heatmap showing the mean expression values (Z-score) for the 139 selected C/T genes in various human adult tissues based on RNA-seq data from GTEx.

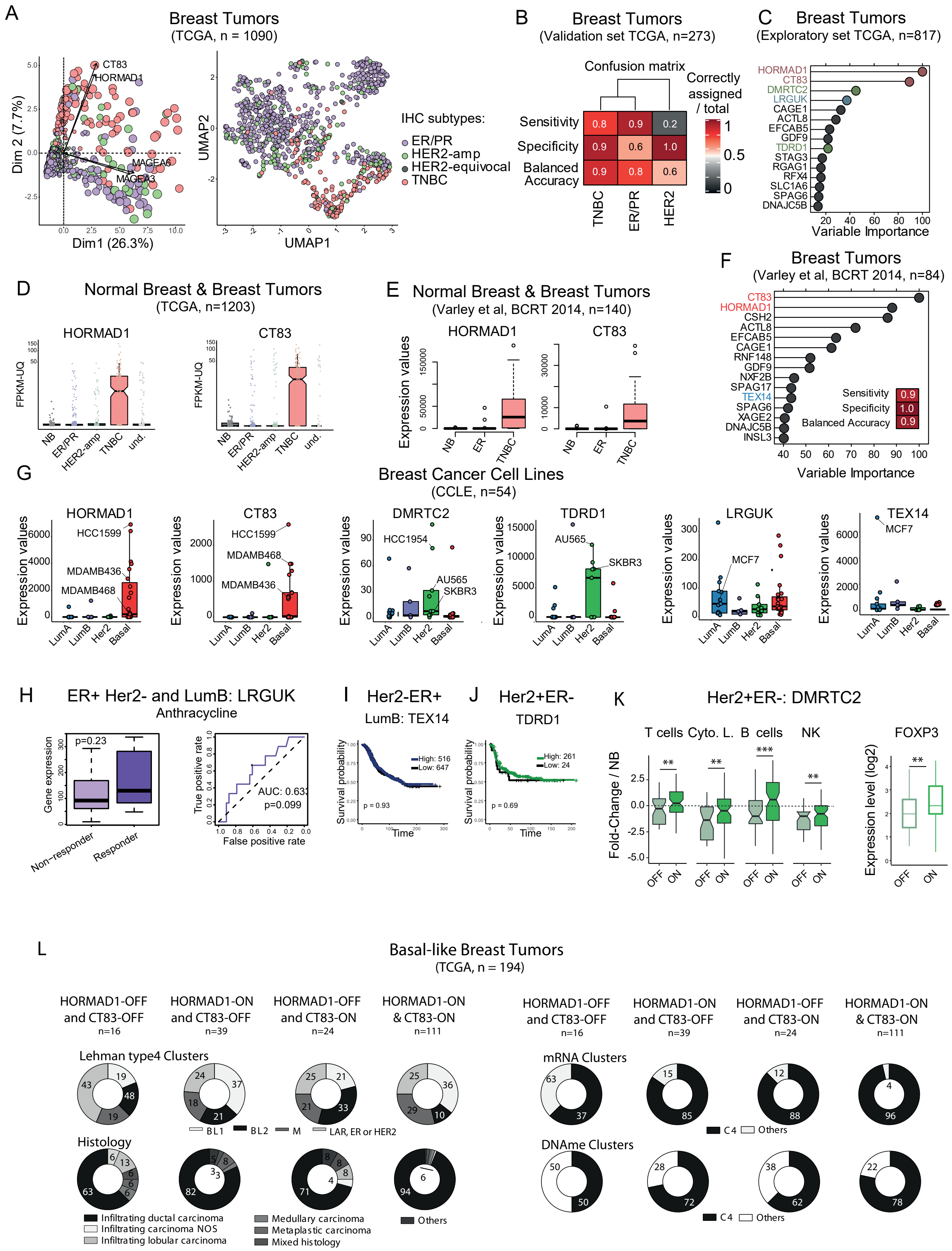

A
Breast Tumors
(TCGA, n = 1090)
Dim 2 (7.7%)
Dim1 (26.3%)
CT83
HORMAD1
MAGEA6
MAGEA3
UMAP2
UMAP1
IHC subtypes:
ER/PR
HER2-amp
HER2-equivocal
TNBC
B
Breast Tumors
(Validation set TCGA, n=273)
Confusion matrix
Correctly assigned / total
Sensitivity 0.8 0.9 0.2
Specificity 0.9 0.6 1.0
Balanced Accuracy 0.9 0.8 0.6
TNBC ER/PR HER2
C
Breast Tumors
(Exploratory set TCGA, n=817)
HORMAD1
CT83
DMRTC2
LRGUK
CAGE1
ACTL8
EFCAB5
GDF9
TDRD1
STAG3
RGAG1
RFX4
SLC1A6
SPAG6
DNAJC5B
Variable Importance
D
Normal Breast & Breast Tumors
(TCGA, n=1203)
HORMAD1
CT83
FPKM-UQ
NB
ER/PR
HER2-amp
TNBC
und.
E
Normal Breast & Breast Tumors
(Varley et al, BCRT 2014, n=140)
HORMAD1
CT83
Expression values
NB
ER
TNBC
F
Breast Tumors
(Varley et al, BCRT 2014, n=84)
CT83
HORMAD1
CSH2
ACTL8
EFCAB5
CAGE1
RNF148
GDF9
NXF2B
SPAG17
TEX14
SPAG6
XAGE2
DNAJC5B
INSL3
Sensitivity 0.9
Specificity 1.0
Balanced Accuracy 0.9
Variable Importance
G
Breast Cancer Cell Lines
(CCLE, n=54)
HORMAD1
CT83
DMRTC2
TDRD1
LRGUK
TEX14
Expression values
HCC1599
MDAMB436
MDAMB468
HCC1954
AU565
SKBR3
MCF7
LumA
LumB
Her2
Basal
H
ER+ Her2- and LumB: LRGUK
Anthracycline
Gene expression
p=0.23
Non−responder
Responder
True positive rate
False positive rate
AUC: 0.633
p=0.099
I
Her2-ER+
LumB: TEX14
Survival probability
High: 516
Low: 647
p = 0.93
Time
J
Her2+ER-
TDRD1
Survival probability
High: 261
Low: 24
p = 0.69
Time
K
Her2+ER-: DMRTC2
T cells
Cyto. L.
B cells
NK
FOXP3
Fold-Change / NB
Expression level (log2)
OFF
ON
L
Basal-like Breast Tumors
(TCGA, n = 194)
HORMAD1-OFF and CT83-OFF n=16
HORMAD1-ON and CT83-OFF n=39
HORMAD1-OFF and CT83-ON n=24
HORMAD1-ON & CT83-ON n=111
Lehman type4 Clusters
BL1
BL2
M
LAR, ER or HER2
Histology
Infiltrating ductal carcinoma
Infiltrating carcinoma NOS
Infiltrating lobular carcinoma
Medullary carcinoma
Metaplastic carcinoma
Mixed histology
Others
mRNA Clusters
DNAme Clusters
C4
Others

Figure S2: Further associations between expression of the 6 discriminating Cancer/Testis genes, tumor properties and survival

A. Multidimensional analysis of TCGA breast tumor and healthy samples based on expression of the 139 selected C/T genes. Each dot represents a sample, the color code corresponds to immunohistochemistry (IHC) classification (based on ER/PR/HER2 expression). Left: Principal Component Analysis, dot sizes are proportional to the quality of representation in PC1/PC2 space. The C/T genes best correlated to PC1/PC2 are represented. Right: Uniform Manifold Approximation and Projection.

B. Confusion matrix for breast tumor samples in the validation cohort (randomly selected 25% samples from the TCGA breast tumors) of the IHC tumor subtypes prediction obtained with the best Random Forest model. This model was established after a 500-tree training on the discovery cohort (the remaining 75%), based on the expression level of the 139 C/T genes.

C. Top 15 most important variables in the best Random Forest model for IHC tumor subtype prediction.

D. Expression levels for the 2 basal-specific C/T genes in the TCGA breast cohort, according to IHC tumor subtype.

E. Expression levels for the 2 basal-specific C/T genes in the Varley et al. breast cohort, according to IHC tumor subtype.

F. Top 15 most important variables in the best Random Forest model for IHC tumor subtype prediction in the Varley et al. cohort.

G. Expression levels for six subtype-specific C/T genes in breast cancer cell lines from the Cancer Cell Line Encyclopedia, according to PAM50 tumor subtype. Some commonly used cell lines are highlighted.

H. Left: Expression value for the luminal-specific C/T gene LRGUK in luminal B tumors, according to the clinical evaluation of tumor response to chemotherapy. Right: ROC curve evaluating the potential of LRGUK as a predictive biomarker of anthracycline chemotherapy response of ER+ Her2- Luminal B tumors.

I. Relapse-free survival curve for Her2-ER+ Luminal B breast cancer patients, as a function of TEX14 expression.

J. Same as in I for TDRD1 expression in Her2+ER- tumors.

K. Immune infiltration of Her2-positive breast tumors that express (ON) or do not express (OFF) DMRTC2, inferred from whole tumor RNA-seq data using MCPcounter. Fold-Changes were computed against Normal Breast (NB). Right: Expression level of the immune suppressive factor FOXP3 in the same tumors. P-value < 0.01: ** ; P-value < 0.001: ***

L. Anatomopathological classification (left) and IntClust classification (right) of basal-like breast tumors from TCGA, according to their expression status of HORMAD1 and CT83 genes.

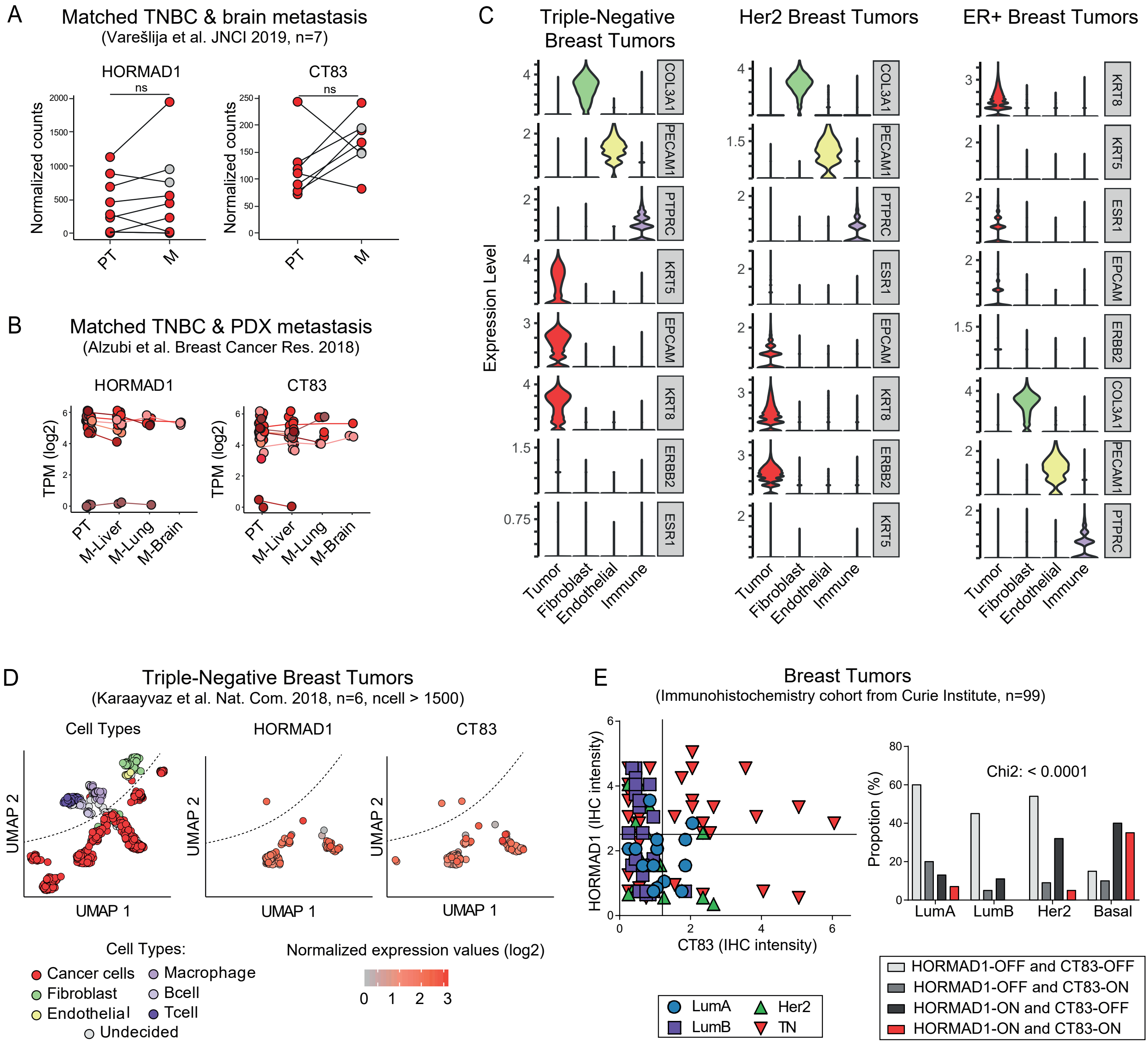

Figure S3: Further characterization of HORMAD and CT83 expression during tumor progression; validation of scRNAseq cluster identities.

A. Normalized expression levels of HORMAD1 and CT83 in matched primary tumors (PT) and metastasis (M).

B. Normalized expression levels of HORMAD1 and CT83 in matched primary tumors (PT) and multiple metastasis (M-) according to the seeding site.

C. ViolinPlot showing the expression of marker genes for each cell type cluster, in scRNA-seq data from different subtypes of breast tumors.

D. UMAP representation of a scRNA-seq study on 6 triple-negative breast tumors (GSE118390). Each dot is either a tumor cell or a cell from the tumor microenvironment. Epithelial clusters of tumor cells are highlighted, HORMAD1 and CT83 expression is shown.

E. Quantification of the IHC signal in breast tumor samples. Subtypes are indicated by the color/ shape code; proportion of positive tumors for HORMAD1 and CT83 according to tumor subtypes are summarized as barplot (right).

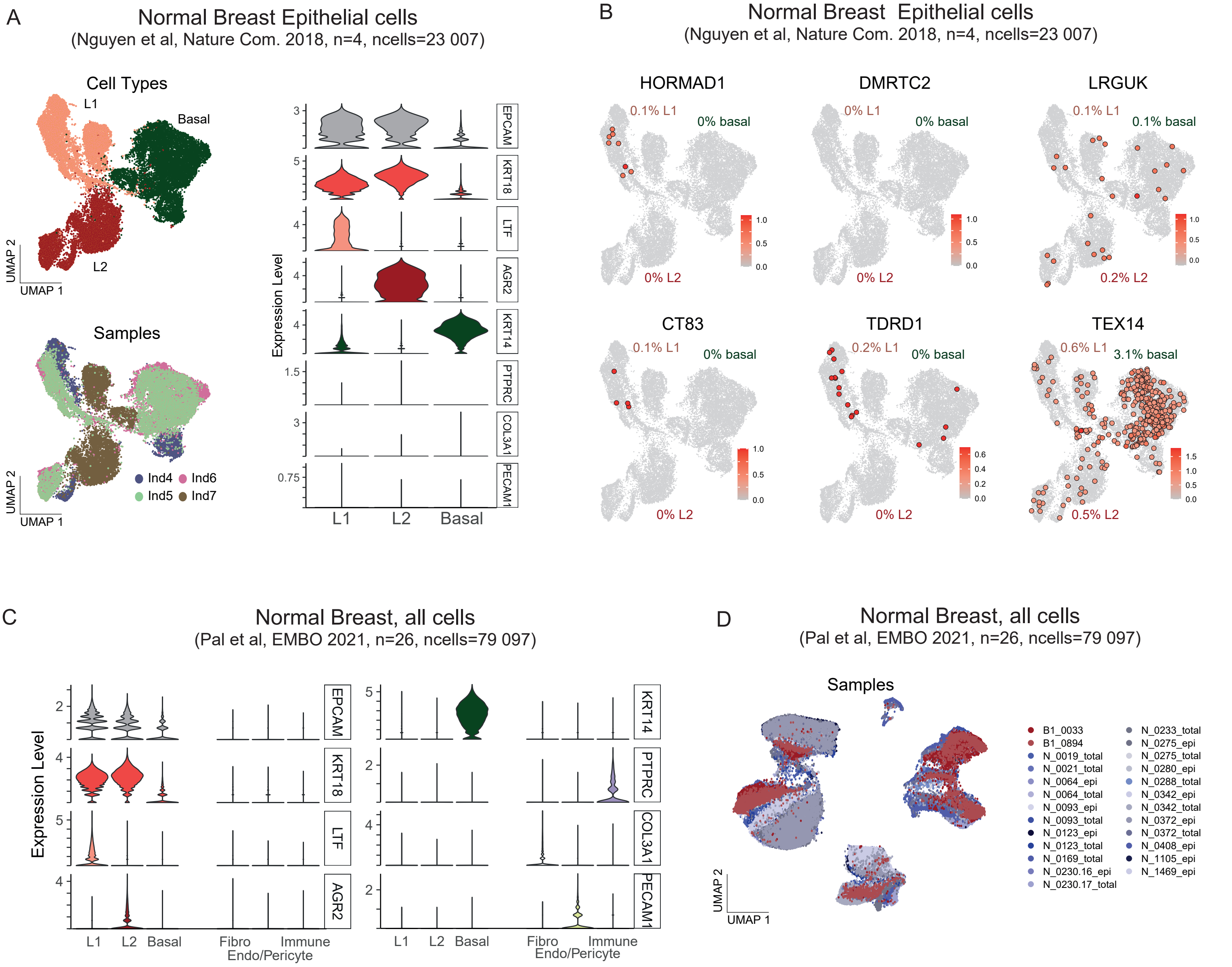

Figure S4: Markers of cell identity in scRNAseq from healthy mammary glands

A. UMAP representation of a scRNA-seq study on 4 healthy mammary glands (GSE113197) after an enrichment of epithelial cells by FACS. Top panel: Cell types. Bottom panel: sample of origin, Ind=Individual. Right: ViolinPlot showing expression values of marker genes for each identified cluster.

B. Same as in A, showing normalized expression of breast-cancer-specific C/T genes. Positive cells are emphasized, the percentage of positive cells in each cluster is indicated.

C. ViolinPlot showing expression values of marker genes for each identified cluster from scRNA-seq of healthy breast samples (GSE161529).

D. UMAP representation of a scRNA-seq study from 26 healthy mammary glands (GSE161529) showing samples of origin. Two prophylactic samples from BRCA1-mutated patients are highlighted in red.

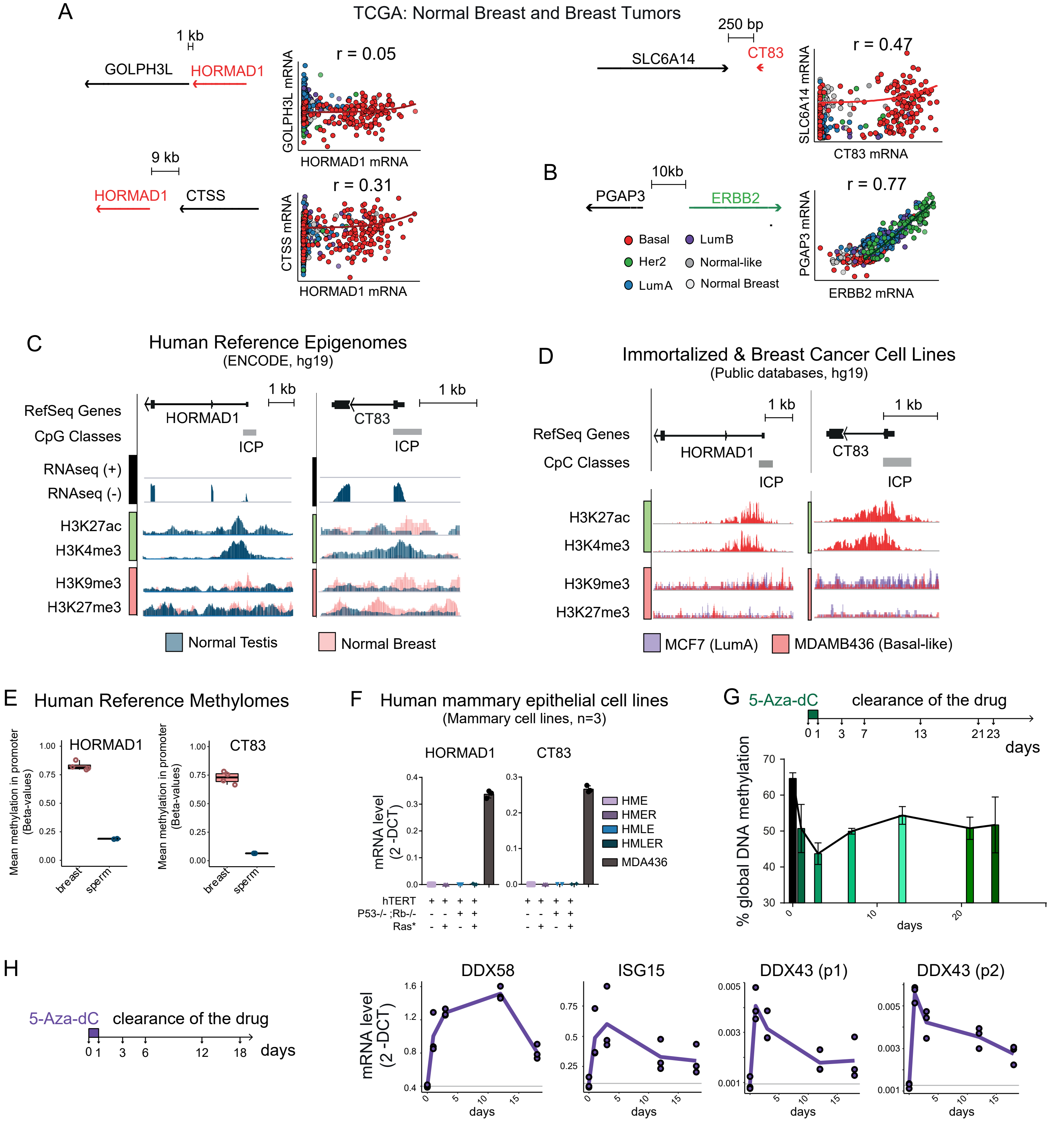

Figure S5: Genetic and epigenetic landscapes of the HORMAD1 and CT83 genes

A. Biplot showing the correlation between HORMAD1 or CT83 expression and expression of the closest neighboring genes. Genomic distances are depicted. The color code corresponds to PAM50 tumor subtypes.
B. Biplot showing the correlation between ERBB2 gene expression (coding for the frequently amplified gene Her2) and the closest neighboring gene, which is frequently co-amplified. Genomic distances are depicted. The color code corresponds to PAM50 tumor subtypes.
C. IGV representation of transcriptomic and histone modification landscapes at HORMAD1 and CT83 loci in healthy Testis and Breast samples (ENCODE).
D. IGV representation of histone modifications landscapes at HORMAD1 and CT83 loci in breast cell lines: MCF7 cells do not express HORMAD1 or CT83, whereas MDA-MB436 cells express both.
E. Mean CpG methylation of CpG probes found in HORMAD1 (left) and CT83 (right) promoters in healthy human breast and sperm samples.
F. RT-qPCR analysis of HORMAD and CT83 expression in human mammary epithelial cells lines after various genetic events and in a basal-like breast cancer cell line (MDA-MB436).
G. Global DNA methylation assessed by LUMA in HMLE cells after a 24-hour treatment with 1uM of 5-aza-dC followed by a 3-week recovery in absence of the drug.
H. mRNA levels of the indicated C/T genes, after a treatment with 5-aza-dC followed by a recovery of 18 days.

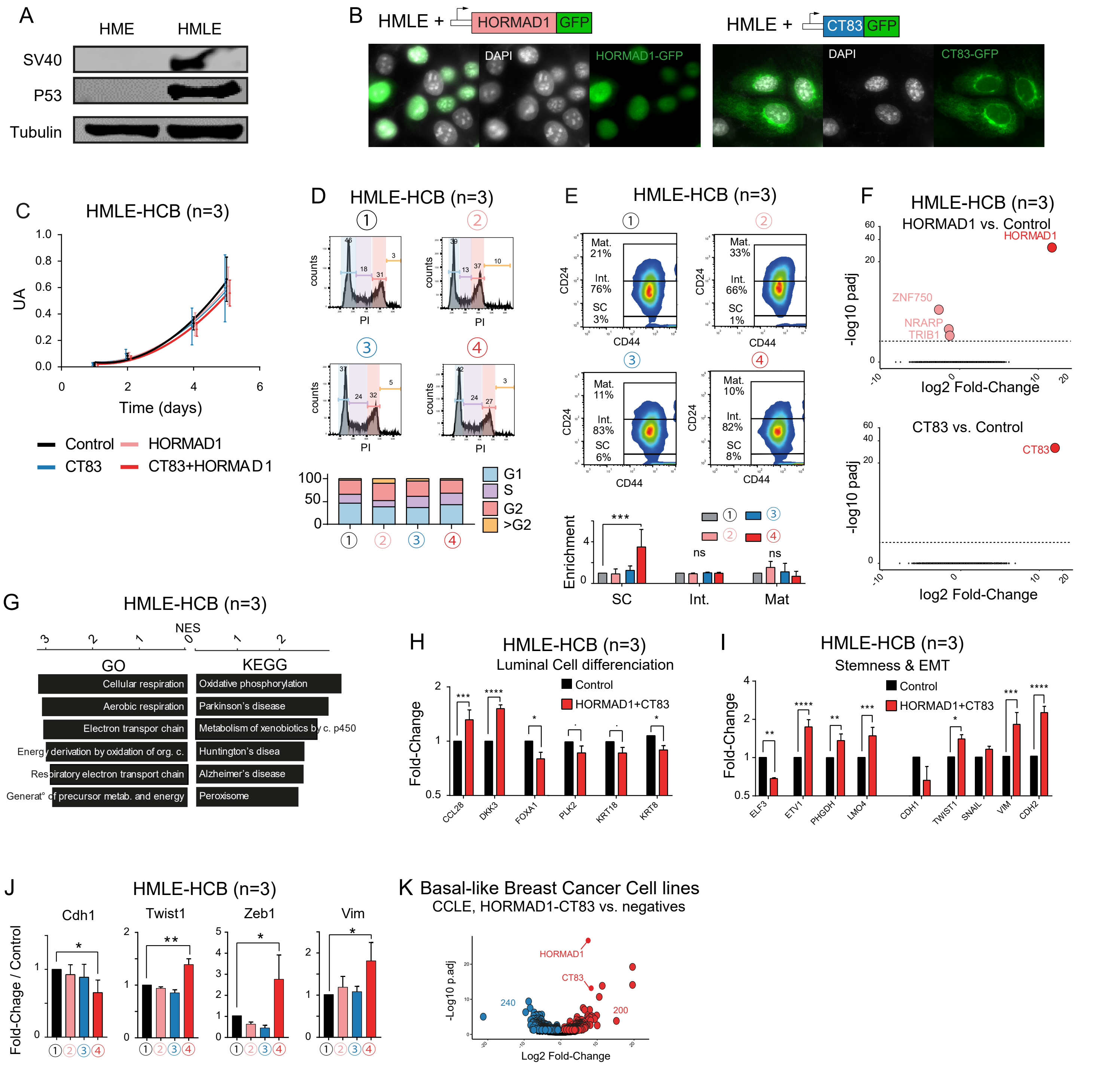

Figure S6: Characterization of HORMAD1 and CT83-expressing cultured breast cells

A. Western Blot showing the expression of transgenic constructs in the HME and HMLE cell lines. B. Immunofluorescence images of HORMAD1-GFP or CT83 in lentivirus-infected cells. Cells were sorted 48h after the infection based on the detection of the GFP signal, and immediately seeded for staining. C. Growth curves of HMLE-derived cell lines, measured by indirect detection of metabolically active cells using the MTS assay (left, mean +/- SD, n=3 independent experiments). D. Cell-cycle analysis of HMLE-derived cell lines. Data are represented as mean +/- SD (n=3 independent experiments) ; a representative FACS plot is shown. E. Flow cytometry analyses showing the percentage of Mature cells (mat, CD44+CD24high), Intermediate cells (int, CD44+CD24low) and Stem cells (SC, CD44+CD24low) in HMLE-derived cell lines. Data are represented as mean +/- SD (n=3 independent experiments) ; a representative FACS plot is shown. F. VolcanoPlot summarizing the differential gene expression analysis in HORMAD1- or CT83- overexpressing HMLE cells versus control HMLE cells. G. Barplot displaying the top 6 pathways differentially activated in HORMAD1 and CT83-positive HMLE-derived cells compared to the control condition, from MSigDB c5_GO and c2_KEGG annotations. X-axis corresponds to -log10 adjusted q-values. H. RT-qPCR analysis of genes related to luminal differentiation in HMLE overexpressing HORMAD1 and CT83 versus control. Data are represented as mean +/- SD (n=3 independent experiments). I. RT-qPCR analysis of genes related to stemness and EMT in HMLE overexpressing HORMAD1 and CT83 versus control. Data are represented as mean +/- SD (n=3 independent experiments). J. RT-qPCR analysis of markers of EMT and regulators of this pathway in HMLE overexpressing HORMAD1 and CT83 versus control. Data are represented as mean +/- SD (n=3 independent experiments). K. Volcano plot of the distribution of differentially expressed genes (p-value adjusted < 0.01) in basal-like breast cancer cell lines (CCLE) according to HORMAD1 and CT83 expression status.

K.
